# Deep linear matrix approximate reconstruction with integrated BOLD signal denoising reveals reproducible hierarchical brain connectivity networks from multiband multi-echo fMRI

**DOI:** 10.3389/fnins.2025.1577029

**Published:** 2025-04-16

**Authors:** Wei Zhang, Alexander Cohen, Michael McCrea, Pratik Mukherjee, Yang Wang

**Affiliations:** ^1^School of Computer and Cyber Sciences, Augusta University, Augusta, GA, United States; ^2^Transdisciplinary Research Initiative in Inflammaging and Brain Aging, Augusta University, Augusta, GA, United States; ^3^Department of Radiology, Medical College of Wisconsin, Milwaukee, WI, United States; ^4^Department of Neurosurgery, Medical College of Wisconsin, Milwaukee, WI, United States; ^5^Department of Radiology and Biomedical Imaging, University of California, San Francisco, San Francisco, CA, United States; ^6^Department of Bioengineering and Therapeutic Sciences, University of California, San Francisco, San Francisco, CA, United States

**Keywords:** deep learning, fMRI, hierarchical brain connectivity networks, multiband, multi-echo, reproducible, resting state

## Abstract

The hierarchical modular functional structure in the human brain has not been adequately depicted by conventional functional magnetic resonance imaging (fMRI) acquisition techniques and traditional functional connectivity reconstruction methods. Fortunately, rapid advancements in fMRI scanning techniques and deep learning methods open a novel frontier to map the spatial hierarchy within Brain Connectivity Networks (BCNs). The novel multiband multi-echo (MBME) fMRI technique has increased spatiotemporal resolution and peak functional sensitivity, while the advanced deep linear model (multilayer-stacked) named DEep Linear Matrix Approximate Reconstruction (DELMAR) enables the identification of hierarchical features without extensive hyperparameter tuning. We incorporate a multi-echo blood oxygenation level-dependent (BOLD) signal and DELMAR for denoising in its first layer, thereby eliminating the need for a separate multi-echo independent component analysis (ME-ICA) denoising step. Our results demonstrate that the DELMAR/Denoising/Mapping strategy produces more accurate and reproducible hierarchical BCNs than traditional ME-ICA denoising followed by DELMAR. Additionally, we showcase that MBME fMRI outperforms multiband (MB) fMRI in terms of hierarchical BCN mapping accuracy and precision. These reproducible spatial hierarchies in BCNs have significant potential for developing improved fMRI diagnostic and prognostic biomarkers of functional connectivity across a wide range of neurological and psychiatric disorders.

## 1 Introduction

Functional Magnetic Resonance Imaging (fMRI) has been widely used to investigate Brain Connectivity Networks (BCNs) (Bartels and Zeki, 2005; Beckmann and Smith, 2005; Biswal et al., 1995, 2010; Bullmore and Sporns, 2009; Duncan, 2010; Stam, 2014). Multiple studies have revealed the hierarchical modular organization of BCNs (Bassett et al., 2008; Biswal et al., 2010; Bullmore and Sporns, 2009; Sporns et al., 2004). The architecture of cortical/subcortical BCNs is organized at multiple spatial scales, from local circuits at the microscale to columns as well as layers at the mesoscale to areas and areal networks at the macroscale (Bullmore and Sporns, 2009; Power et al., 2011; Stam, 2014; Sporns et al., 2004). Notably, BCNs, which integrate spatial structure with brain functionality, usually reveal specific brain regions associated with distinct functions. This approach has established more straightforward analytics than traditional examinations on sequential time-series (Agarwal et al., 2023; Peng et al., 2023; Stam, 2014). For example, during the COVID-19 pandemic, researchers identified impaired BCNs including olfactory cortex in patients experiencing olfactory loss, highlighting specific neural disruptions linked to certain symptoms (Wingrove et al., 2023).

In the past two decades, fMRI acquisition techniques, such as multiband (MB) and multi-echo (ME) echoplanar imaging (Cohen et al., 2018, 2020; Cohen et al., 2021a,b), and computational approaches for fMRI analytics, e.g., General Linear Modeling (GLM), Graph Theory, Independent Component Analysis (ICA), and Sparse Dictionary Learning (SDL) (Andersen et al., 1999; Calhoun et al., 2001; Lee et al., 2011; Lee et al., 2016; Lv et al., 2015a,b; Zhang et al., 2017; Zhang et al., 2018a,b), have been proposed to map BCNs. However, most analytic methods depend on a ‘shallow’ architecture that cannot detect the spatial hierarchy and overlapping structures of BCNs in an unsupervised data-driven fashion using resting-state fMRI (rsfMRI) or task-based fMRI (tfMRI) signals (Hu et al., 2018; Huang et al., 2018; Zhang et al., 2019, 2020a). Some spatial hierarchies in BCNs have been identified via shallow linear models, such as ICA (Iraji et al., 2019; Smith et al., 2009; Wylie et al., 2021), but there is no principled way to map this hierarchical organization using shallow methods.

Fortunately, the rapid development of deep learning methods provides an opportunity to identify hierarchical BCNs. For example, the dimensional reduction/low-order method (Wylie et al., 2021) is a data-driven deep method that extracts hierarchical meta-BCNs (e.g., BCNs identified at deep layers) organized as several entire/partial areas of other BCNs, e.g., twelve canonical BCNs (Smith et al., 2009). These canonical BCNs typically represent fundamental brain functions, such as auditory, visual, and olfactory processing (Smith et al., 2009). Notably, these canonical BCNs usually are identified at shallow layers of deep learning methods (Esteva et al., 2019; Zhang et al., 2019, 2020a,b). Meanwhile, a wide array of deep learning methods enable the reconstruction of hierarchical architectures in BCNs, including the Deep Convolutional Auto Encoder (DCAE), Deep Belief Network (DBN), and Convolutional Neural Network (CNN) (Bengio et al., 2012; Esteva et al., 2019; Gurovich et al., 2019; Hannun et al., 2019; LeCun et al., 2015; Plis et al., 2014; Schmidhuber, 2015; Suk et al., 2014; Suk et al., 2016; Topol, 2019; Zhang et al., 2020a). Compared with these deep nonlinear models (e.g., deep neural networks), the recently proposed DEep Linear Matrix Approximate Reconstruction (DELMAR) has several advantages (Zhang and Bao, 2022): (1) training samples as small as a single subject (Hinton et al., 2012); (2) less extensive computational resources, e.g., central processing units (CPUs) (Bengio et al., 2012; Hinton et al., 2006; Hinton et al., 2012; Huang et al., 2018; Zhang et al., 2020a,b); (3) automatic tuning of hyperparameters, including the number of layers and size of the weight matrix (Ilievski et al., 2017; Zhang et al., 2020b) [currently, the determination of the number layers and the number of BCNs at each layer remains arbitrary (Pfob et al., 2022)]; (4) a less time-consuming training process (Esteva et al., 2019); and (5) a guarantee to converge to the unique fixed point (Esteva et al., 2019; Gurovich et al., 2019; Hannun et al., 2019). Furthermore, a previous simulation study (Zhang et al., 2020b) has already proven that DELMAR can successfully recognize hierarchical BCNs. Integrating the more sensitive and specific multiband multi-echo (MBME) fMRI techniques (Cohen et al., 2020) with DELMAR can provide an insightful opportunity to further explore the hierarchical structures of BCNs (Zhang et al., 2020a,b). Having previously validated DELMAR through an in-silico fMRI approach in experimental studies comparing it to other peer methods (Qiao et al., 2021; Wylie et al., 2021; Trigeorgis et al., 2014; Trigeorgis et al., 2016; Zhang et al., 2020b, 2024; Zhang and Bao, 2022), we are now particularly interested in exploring the reproducibility of hierarchical BCNs identified via DELMAR and assessing its potential denoising capabilities.

Notably, state-of-the-art MBME fMRI has been proven to increase spatial and temporal resolution, enhance signal-to-noise ratio (SNR) in fMRI signals, and improve functional sensitivity (Cohen et al., 2020, 2021a,b). As we navigate the era of deep learning, advanced methods have paved the way to reveal more reproducible hierarchical brain connectivity networks (BCNs). Therefore, on the one hand, we aim to apply DELMAR to investigate whether we can further identify more reproducible hierarchical spatial functional connectivity mapping from the MBME technique compared to canonical MB fMRI. On the other hand, inspired by previous work (Zhang et al., 2020a), we aim to explore the potential capability of DELMAR to perform denoising at shallow layers instead of traditional high-pass filtering. To investigate the denoising capability of DELMAR at shallow layers, we developed two computational frameworks: (1) ME-ICA & DELMAR, where DELMAR is applied to MBME resting-state fMRI denoised by the ME-ICA, and (2) DELMAR/Denoise/Mapping, where DELMAR is directly applied to the raw data of resting-state MBME fMRI. Moreover, test–retest scans are investigated with the following hypotheses: (a) MBME fMRI will reveal more reproducible hierarchical BCNs than MB fMRI. (b) DELMAR/Denoise/Mapping will produce more reproducible results than ME-ICA & DELMAR in lower- and medium-level BCNs. Previous work has shown that independent constraints could disrupt some spatially overlapped regions in BCNs, and ICA may smooth various strongly overlapped areas (Zhang et al., 2018a,b). We aim to further validate the performance of DELMAR in both denoising and BCN identification. (c) DELMAR/Denoise/Mapping will yield more reproducible results than ME-ICA & DELMAR in high-level BCNs.

## 2 Materials and methods

### 2.1 ME-ICA & DELMAR verses DELMAR/Denoise/Mapping for deriving hierarchical brain connectivity networks

**Figure 1:**
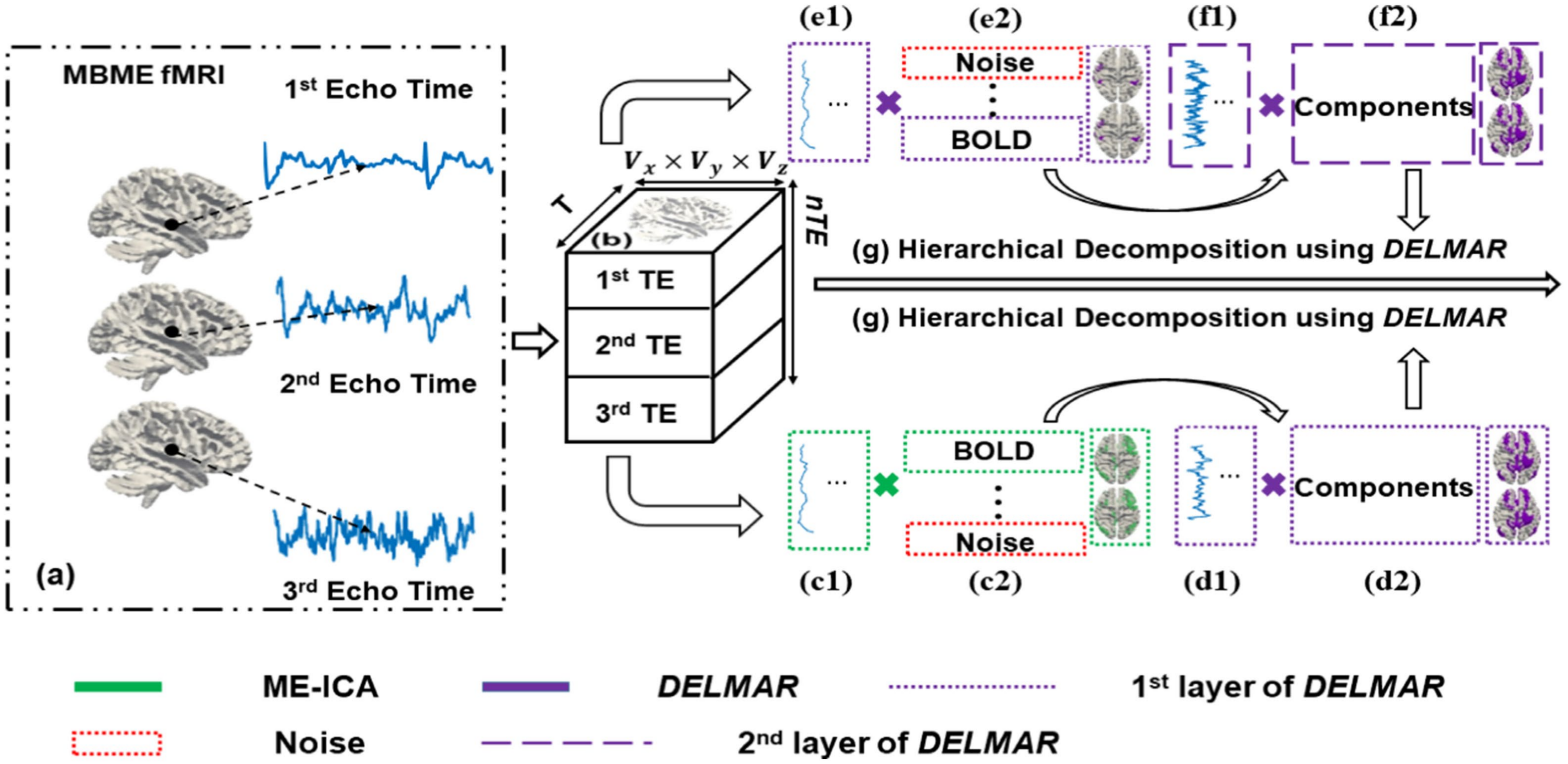
ME-ICA & DELMAR vs. DELMAR/Denoise/Mapping. This figure describes two computational frameworks proposed in this work to extract hierarchical BCNs. **(a)** S represents the input MBME fMRI signal matrix with three multi-echo times, e.g., 1st to 3rd Echo Time; for a single echo time, it contains the T time points and M voxels. **(b)** Reorganize the MBME fMRI data as a three-dimensional signal matrix. **(c)** Describes the pipeline of a ME-ICA to denoise in which the original input signal is decomposed into the weight matrix (shown as **c1**) and independent component matrix, i.e., functional connectivity networks (shown as **c2**). **(d,e)** Represent the first layers of DELMAR. **(d1)** Represents the first layer weight matrix/dictionary of DELMAR identified from Blood Oxygen-Level Dependent (BOLD) components. **(d2)** Represents the first layer feature matrix of DELMAR, i.e., connectivity networks recognized via BOLD components. Similarly, **(e1,e2)** represent the corresponding matrices of the first layer of DELMAR, which are considered to play the role of denoise. **(f1,f2)** Represent the corresponding matrices of derived BOLD components from the first layer feature matrix. **(g)** Demonstrates the hierarchical decomposition of the previous feature matrix to reveal the high-level BCNs, for both computational frameworks. The dashed line indicates the different layers of DELMAR; meanwhile, the rectangle in green and purple represents the method ME-ICA and DELMAR, respectively.

The following section introduces the fundamental descriptions of each computational framework used to extract hierarchical BCNs. Furthermore, these descriptions are prerequisites to analyzing the properties of each model in the succeeding sections. Overall, in Figure 1, the computational steps of ME-ICA & DELMAR vs. DELMAR/Denoise/Mapping that is based on the deep linear methods are outlined. Specifically, Figure 1 demonstrates two computational frameworks adopted in this study: (1) ME-ICA & DELMAR employs ME-ICA (Kundu et al., 2012) to denoise first, followed by DELMAR to extract hierarchical BCNs; (2) DELMAR is directly used to denoise and map the hierarchical BCNs. Since ME-ICA is a vital technique in fMRI research, the comparisons between ME-ICA and DELMAR will further validate the innovative method DELMAR and investigate the reproducibility of revealed hierarchical BCNs.

### 2.2 Multi-echo independent component analysis for denoising

ICA is usually employed to analyze fMRI signals (Calhoun et al., 2001). With ME fMRI, BOLD contrast optimization can be achieved by combining time series of various TEs using a weighting scheme to better map functional connectivity in the human brain (Kundu et al., 2012). These contrast optimizations benefit in removing potential artifacts and thermal noise (Posse et al., 1999). Thermal noise is usually identified as a significant source of signal fluctuations at clinical field strengths, especially when high receiver bandwidths and/or high spatial resolutions are used. Thus, considering the efficacy of ICA, an ME-ICA was proposed to denoise the ME fMRI signal by Kundu et al. (2012).

To separate time series that are common across all TEs, i.e., BOLD signal, and thermal noise, TEs were treated as a three-dimensional spatial matrix for spatial ICA (Kundu et al., 2012). The MBME fMRI data were decomposed as described in the following Equation 1:


(1) $$\begin{array}{c} S\underline{\underline{def}}\left[T,V_{x}\times V_{y}\times V_{z}\mathrm{,nTE}\right]\\ S\overset{ICA}{\to }\left[\mathrm{T,IC}\right]\times \left[{\mathrm{IC,V}}_{x}\times V_{y}\times V_{z}\mathrm{,nTE}\right] \end{array}$$

where S denotes the time series of MBME fMRI signal; $V_{x}$ , $V_{y}$ , and $V_{z}$ are the coordinates of voxels within the functional brain mask (Lv et al., 2017); T is the total number of time points; nTE is the number of echoes; IC is the number of components; $\left[\text{.}\right]$ denotes a matrix. For example, $\left[T,V_{x}\times V_{y},V_{z}\times nTE\right]$ denotes a ME fMRI signal matrix that is organized as a three-dimensional matrix. Its size is the total number of time points T , a two-dimensional single slice of functional brain mask $V_{x}\times V_{y}$ , and the total number of spatial components of multi TEs, $V_{z}\times nTE$ . Then, applying spatial ICA on the MBME fMRI signal matrix S , BOLD, and noise components can be almost separated (Kundu et al., 2012; Cohen et al., 2020). Notably, the ME-ICA technique takes into consideration both kappa, which represents TE-dependence, and rho, indicative of TE-independence, when separating non-BOLD components from BOLD signals (Cohen et al., 2021a,b). The K spectrum clustering method is also employed to differentiate the BOLD and noise components. This approach allows for more precise separation of true neural activity from physiological noise and other artifacts, enhancing the reliability of fMRI data analysis.

Furthermore, inspired by previous works (Zhang et al., 2020a,b), using an $\ell_{1}$ norm penalty (illustrated as ${Z_{i}}_{1}$ in Equation 2) is reasonable for performing spatial denoising on background components. Since noise components are usually weak patterns, a sparse representation using the $\ell_{1}$ norm penalty can continuously remove these weak patterns throughout the iterations. Moreover, Figures 1c2,e2 qualitatively depict the denoising process of DELMAR. For instance, most noisy components, which are relatively weak patterns, will be continuously eliminated via the $\ell_{1}$ norm penalty (Ling et al., 2023). Moreover, using Rank Reduction Operator (RRO) technology, noise components, which usually share small similarity (calculated via Equations 6, 7) with other major components (Wen et al., 2012; Shen et al., 2014), can be easily removed during rank reduction. To summarize, when employing DELMAR, a natural idea to bypass the ME-ICA denoising step is to use the first layer of DELMAR to perform the denoising and then the denoised MBME fMRI can be considered as an input signal for subsequent layers of DELMAR to perform hierarchical spatial decomposition.

### 2.3 DELMAR for denoising and mapping hierarchical BCNs

In this study, we concentrate on the exploration of reproducible spatially hierarchical BCNs using DELMAR and MBME fMRI. As discussed, due to several reported superiorities of DELMAR (Zhang et al., 2020b, 2024; Zhang and Bao, 2022), DELMAR was used to detect hierarchical BCNs. The DELMAR approach employs linear matrix decomposition and a sparse denoising operator. By incorporating an additional dimension to its first layer to accommodate multiple echo times, DELMAR can also perform integrated multi-echo BOLD denoising that is comparable to the ME-ICA.

Therefore, we propose a novel computational framework that performs integrated BOLD denoising and extracting hierarchical features using DELMAR. Specifically, the first layer of DELMAR can denoise the BOLD signal, replacing the ME-ICA in the previous frameworks introduced in Section 2.2. Furthermore, due to the similar method of low-rank estimation, DELMAR can also rank the identified components as ICA does. The noise component(s) is(are) thus automatically ranked at the lower order, and deeper layers can continuously implement the extraction of hierarchical BCNs.

The equation governing DELMAR for a three-dimensional input signal matrix is:


(2) $$\begin{array}{c} \min_{Z_{i}\in {\mathbb{R}}^{m\times n\times r}}\;\overset{M}{\underset{i=1}{\cup }}{Z_{i}}_{1}\\ s.t.\overset{M}{\underset{i=1}{\prod }}X_{i}Y_{M}+Z_{M}=S\\ X_{i}Y_{i}\leftarrow R\left(Y_{i-1}\right),\forall 2\leq i\leq M \end{array}$$

where ${\left\{X_{i}\right\}}_{i=1}^{M}$ represents the hierarchical weight matrices, e.g., $X_{i}$ indicates the matrix of the i th layer. M is the total number of layers. Similarly, ${\left\{Y_{i}\right\}}_{i=1}^{M}$ represents the hierarchical spatial features, e.g., $Y_{i}$ indicates the spatial features of i^th^ layer. ${\left\{Y_{i}\right\}}_{i=1}^{M}$ is also denoted as a correlation matrix, i.e., components matrix. ${\left\{Z_{i}\right\}}_{i=1}^{M}$ are the matrices of background components, which is usually treated as the noise components, due to these matrices are sparse. Importantly, ${\left\{X_{i}\right\}}_{i=1}^{M}$ , ${\left\{Y_{i}\right\}}_{i=1}^{M}$ , and ${\left\{Z_{i}\right\}}_{i=1}^{M}$ are 3D matrices, since input multi-echo fMRI signal matrix is a 3D matrix (Kundu et al., 2012; Cohen et al., 2020). In addition, ℛ represents a rank reduction operator (RRO) to automatically estimate the hyperparameters and more details has been introduced in our previous research work (Zhang et al., 2020b). Naturally, we assume the spatial features $Y_{i-1}$ can be decomposed as deeper dictionary $X_{i}$ and spatial features $Y_{i}$ , in order to implement the deep linear framework (Figure 1). Therefore, the original input data SG can be decomposed as $\overset{M-1}{\underset{i=1}{\prod }}X_{i}Y_{M}$ . In Equation 1, the sparse trade off ${Z_{M}}_{1}$ to control the sparsity levels of background components is determined by $\frac{1}{\beta }$ . And β>0 is a penalty parameter introduced in the following Augmented Lagrangian Function of Equation 2. And S denotes the input signal matrix that is the same in Equation 1 (Zhang et al., 2020b). Notably, using $\ell_{1}$ norm penalty (Liu et al., 2010), ${Z_{i}}_{1}$ represents performing sparse representation on background matrix (Ling et al., 2023), which denoising these noise components within all iterations.

Briefly, the optimization function shown in Equation 2 consists of more variables than conventional methods. Therefore, before optimizing Equation 2, we need to convert Equation 2 to an augmented Lagrangian function. Considering the kth layer, we have the following:


(3) $$L_{\beta }\left(\overset{k}{\underset{i=1}{\prod }}X_{i},Y_{k},Z_{k},e_{k}\right)\underline{\underline{def}}\frac{\beta }{2}{\left\|\overset{k}{\underset{i=1}{\prod }}X_{i}Y_{k}-S\right\|}_{F}^{2}+\langle \overset{k}{\underset{i=1}{\prod }}X_{i}Y_{k}-S,e_{k}\rangle +\frac{1}{\beta }{\left\|Z_{k}\right\|}_{1}$$

Since the Augmented Lagrangian Function shown in Equation 3 is not jointly convex, for the k^th^ layer (we assume the total number of layers is k), a crucial approach has been to use alternating minimization (Jain et al., 2013). Previous works (Jain et al., 2013; Lee and Seung, 1999; Nishihara et al., 2015; Shen et al., 2014; Wen et al., 2012) inspired us to employ ADMM to implement alternative optimization. To solve ${\left\{Z_{i}\right\}}_{i=1}^{M}$ , we jointly utilize the shrinkage method. In Equation 3, all parameters are as discussed before, with $e_{k}$ defined as the multiplier of ADMM. The $\ell_{1}$ norm of $Z_{k}$ shown in Equation 2 can be solved directly using the shrinkage method (Beck and Teboulle, 2009). The convergence of ADMM has been proved as geometric with linear convergence (Jain et al., 2013; Nishihara et al., 2015). Naturally, it is easier to comprehensively employ alternative optimizer and shrinkage methods (Wen et al., 2012). The computational frameworks using DELMAR to denoise MBME fMRI at the first layer are shown below:


(4) $$S\overset{\mathrm{DELMAR}}{\to }\left[{T,D}_{1}\right]\times \left[D_{1},V_{x}\times V_{y}\times V_{z}\mathrm{,nTE}\right]$$

where $D_{1}$ represents the number of extracted potential BOLD components at the 1^st^ layer. Other variables are defined the same as Equation 2. Since DELMAR has a similar performance of low-rank decomposition, it is automatically ranking the principal components like Principal Component Analysis (PCA). Meanwhile, a sparse operator can perform denoising simultaneously. Thus, DELMAR can denoise continuously layer by layer. The further hierarchical decomposition is shown as:


(5) $$\begin{array}{c} \left[D_{1},V_{x}\times V_{y},V_{z}\times TE\right]\overset{\mathrm{DELMAR}}{\to }\left[D_{1},D_{2}\right]\times \left[D_{2},V_{x}\times V_{y}\times V_{z}\mathrm{,nTE}\right]\\ \left[D_{2},V_{x}\times V_{y},V_{z}\times TE\right]\overset{\mathrm{DELMAR}}{\to }\left[D_{2},D_{3}\right]\times \left[D_{3},V_{x}\times V_{y}\times V_{z}\mathrm{,nTE}\right]\\ \begin{array}{c} \vdots \\ \left[D_{k-1},V_{x}\times V_{y},V_{z}\times TE\right]\overset{\mathrm{DELMAR}}{\to }\left[D_{k-1},D_{k}\right]\times \left[D_{k},V_{x}\times V_{y}\times V_{z}\mathrm{,nTE}\right] \end{array} \end{array}$$

where ${\left\{D_{k}\right\}}_{k=1}^{M}$ represents the size of hierarchical weight matrices, e.g., $D_{i}$ indicates the matrix of the i th layer. Compared with Equations 1, 5 provides a computational framework to directly utilize DELMAR to extract spatial features, e.g., BCNs. If considering Equations 4, 5, this combination demonstrates the direct utilization of DELMAR for both denoising (implemented in the 1^st^ layer) and hierarchical feature extraction (implemented in deeper layers).

Notably, to automatically determine the size and number of layers, a vital technique named RRO is introduced (Zhang et al., 2020b). Briefly, RRO focuses on the identification of major components included in the raw data and simultaneously determines which components are relatively weak and that will therefore be continuously merged into the background matrices. In general, RRO demonstrates that the number of units, i.e., layer size, should be consistently reduced if considering deeper layers (Hinton et al., 2012; Zhang et al., 2019, 2020a,b). In other words, the continuous increase of units in deeper layers can result in a lack of convergence. If the number of units/dictionary size, i.e., the estimated rank of the matrix, is reduced to one, that indicates the decomposition should be terminated. Hence, the layer that owns a rank of unity should be considered the final layer. DELMAR employs RRO to continuously reduce the dictionary size and therefore also determine the number of layers. In fact, it does not require any manual design for the essential hyperparameters of deep learning, such as the number of layers or units in each layer used in DBN and other peer deep learning methods.

In detail, this rank estimator RRO employs a technique of rank-revealing by continuously using orthogonal decomposition, in this case via QR factorization (Wen et al., 2012; Shen et al., 2014). The advantage of QR is that it is faster and makes fewer requirements of the input matrix. For example, QR performs orthogonal decomposition faster than Singular Value Decomposition (SVD) and can solve incomplete and over-complete problems.

Initially, we assume that r^*^ is denoted as the initially estimated rank of $S^{i}$ and we denote r as the optimal rank estimation of the input matrix $S^{i}$ . If r^*^ ≥≥ r holds, the detection of the diagonal line of the upper-triangular matrix in the QR factorization can be performed using the input matrix $S^{i}$ . If we can determine the ideal size of QR factorization using $S^{i}$ in the work with permutation matrix and the diagonal matrix R is non-increasing in magnitude (Wen et al., 2012; Shen et al., 2014). The QR factorization and rank-revealing will eventually provide a reasonable solution using a proper thresholding value introduced in Equations 2, 3 (Wen et al., 2012; Shen et al., 2014). Along the main diagonal of matrix R, the weighted ratio (WR) and weighted difference (WD) are used to estimate the rank as follows:

If we denote $d\in {\mathbb{R}}^{r}$ $d\in R^{k}$ and $r\in {\mathbb{R}}^{r-1}r\in R^{k-1}$ , WR can be calculated by Equation 6:


(6) $$\begin{array}{c} d_{i}\leftarrow |R_{ii}|\\ {wr}_{i}\leftarrow \frac{d_{i}}{d_{i+1}} \end{array}$$

where $R_{ii}$ represents a diagonal element of matrix R derived by QR decomposition and ${wr}_{i}$ denotes a single value of WR. The value of each WR is calculated by the ratio of the current element of the diagonal and the following element.

WR is calculated as:


(7) $${wd}_{i}\leftarrow \frac{\left|d_{i}-d_{i-1}\right|}{\sum_{k=1}^{i-1}d_{k}}$$

WR is the difference of the current diagonal element and the previous one divided by the cumulative sum of all previous diagonal elements.

Besides, Weighted Correlation (WC) is described as follows:


(8) $${wc}_{i}\leftarrow \frac{\left|\mathrm{corr}\left(D_{i+2},D_{i+1}\right)-\mathrm{corr}\left(D_{i+1},D_{i}\right)\right|}{\sum_{q=1}^{i-1}\left\|D_{q}\right\|}$$

Notably, Equation 8 calculates the absolute correlation differences between adjacent components, such as $D_{i-2}$ , $D_{i-1}$ , and $D_{i}$ represent the ${\left(i-2\right)}^{th}$ , ${\left(i-1\right)}^{th}$ , and $j^{th}$ row in the decomposed matrix $V^{T}$ , $i\in \left[1,m-2\right]$ . In particular, $\mathrm{corr}\left(.,\text{.}\right)$ represents a correlation of two BCNs. Thus, RRO can iteratively determine the estimated layer size, e.g., the number of BCNs at each layer.

Since WD, WR, and WC are the cumulative ratio, difference, the correlation of adjacent components, and the number of components, e.g., BCNs, can be reduced by at least one in each iteration. Thus, the RRO iteratively utilizing WR, WD, and WC can guarantee convergence (Zhang et al., 2020b). Importantly, as previously introduced, noise components usually share smaller spatial correlations, such as ${wr}_{i}$ , ${wd}_{i}$ , and ${wc}_{i}$ with other components. Thus, these noise components can be continuously removed during rank reduction when reducing the dimensionality of the component matrix.

The mathematical definition of RRO is described in Equations 9, 10 as below:


(9) $$ℛ\left[\begin{array}{c} a_{1}\\ a_{2}\\ \begin{array}{c} \vdots \\ a_{n-1}\\ a_{n} \end{array} \end{array}\right]=\left[\begin{array}{c} {a_{1}}^{\left(1\right)}\\ {a_{2}}^{\left(1\right)}\\ \begin{array}{c} \vdots \\ {a_{n-2}}^{\left(1\right)}\\ {a_{n-1}}^{\left(1\right)} \end{array} \end{array}\right]\;{ℛ}^{k}\left[\begin{array}{c} a_{1}\\ a_{2}\\ \begin{array}{c} \vdots \\ a_{n-1}\\ a_{n} \end{array} \end{array}\right]=\left[\begin{array}{c} {a_{1}}^{\left(1\right)}\\ {a_{2}}^{\left(1\right)}\\ \begin{array}{c} \vdots \\ {a_{n-k-1}}^{\left(1\right)}\\ {a_{n-k}}^{\left(1\right)} \end{array} \end{array}\right]$$

where ℛ denotes the RRO operator; and theoretically, if k is large enough, we have ${ℛ}^{k}\left[a_{1},a_{2},\cdots ,a_{n}\right]=\left[\hat{a\;}\right]$ . Also, if $\mathrm{rank}\left(\left[\hat{a\;}\right]\right)=1$ , k is equivalent to the total number of layers. These clearly demonstrate that RRO can continuously reduce the dimensions of the original data and retain the vital components that is comparable to robust PCA (Jain et al., 2013). By continuously using low-rank estimation, DELMAR implements the automatic estimation of dictionary size and number of layers.

### 2.4 Pre-processing of resting-state MB and MBME fMRI data, ground truth templates and methodological hyperparameters tuning

This study was approved by the Medical College of Wisconsin Institutional Review Board and was conducted in accordance with the Declaration of Helsinki. All subjects provided written informed consent prior to participation in this study. In total, 28 healthy volunteer subjects (Mean Age = 28.0 y.o., Range 20–46 y.o., 9 Male, 19 Female) participated in this study. Of those, 19 subjects returned (Mean Age = 27.2 y.o., Range 20–46 y.o., 7 Male, 12 Female) within 2 weeks to repeat the study. Subjects were instructed to refrain from caffeine and tobacco for 6 h prior to imaging.

Specifically, the maximum gradient strength was 70 mT/m, and the maximum slew rate was 170 mT/m/ms. Each subject underwent two resting-state fMRI (rsfMRI) acquisitions: an MB scan and an MBME scan. The MB scan had the following parameters: TR/TE = 650/30 ms, FOV = 24 cm, matrix size = 80 × 80 with slice thickness = 3 mm (3 × 3 × 3 mm voxel size), 11 slices with a multiband factor of 4 (44 total slices), FA = 60°, and partial Fourier factor = 0.85. The MBME scan had the following parameters: TR/TE = 900/11, 30, 49 ms, FOV = 24 cm, matrix size = 80 × 80 with slice thickness = 3 mm (3 × 3 × 3 mm voxel size), 11 slices with a multiband factor of 4 (44 total slices), FA = 60°, and partial Fourier factor = 0.85. Both scans used an EPI readout with in-plane acceleration (R) = 2. The resting-state scans lasted 6 min each, resulting in 554 volumes for the MB scans and 400 volumes for the MBME scans. During the resting-state scans, subjects were instructed to close their eyes but remain awake, refrain from any motion, and not think about anything in particular (Cohen et al., 2020). Notably, recent research by Han et al. (2023) suggests that eyes closed tend to correlate with greater integration, while eyes open correlate with greater specialization (Han et al., 2023). In this work, to advance the identification of meta-BCNs, an integration of multiple shallow BCNs, we recommended that all participants keep their eyes closed.

The MB fMRI data preprocessing followed the steps in Lv and Smith’s works (Lv et al., 2017; Smith et al., 2013). The preprocessing pipelines generally included skull removal, motion correction, slice time correction, spatial smoothing, and global drift removal (high-pass filtering). Finally, a brain mask is applied to extract all fMRI signals. Notably, due to the limitations of ICA demonstrated in previous work (Zhang et al., 2018a,b), we have decided to employ the preprocessing pipeline proposed in the works of Lv et al. (2017) and Smith et al. (2013) to reduce the influence of ICA in separating meta-BCNs at deep layers into independent patterns, which results in missing meta-BCNs at deep layers.

Meanwhile, unfortunately, the preprocessing pipeline proposed by Lv et al. (2017) and Smith et al. (2013) cannot be applied to preprocess multi-echo fMRI, such as MBME. Importantly, considering the technical limitations of ICA (Zhang et al., 2018a,b), it is more challenging for MBME fMRI to provide more reproducible meta-BCNs after denoising using ICA. Therefore, the MBME fMRI data preprocessing follows the steps outlined in the works of Kundu et al. (2012), Cohen et al. (2020), Kundu et al. (2012), and Cohen et al. (2020). Briefly, the anatomical T1-weighted image was AC/PC aligned and non-linearly registered to MNI space. Thus, the volume of the MBME fMRI data was registered, e.g., MBME images of multi-echo are registered to MNI standard space. Then, the data were denoised using ME-ICA. This ICA-based pipeline employs a clustering method to separate independent components, specifically BOLD versus non-BOLD components, based on whether their amplitudes are linearly dependent on TE (Kundu et al., 2012; Olafsson et al., 2015). Non-BOLD components were separated from the combined ME data, resulting in a denoised dataset. Notably, to mitigate the potential effects of head motion in MB and MBME fMRI data, we calculated framewise displacement (FD) using fsl_motion_outliers in FSL. The comparison between MB and MBME scans revealed no statistically significant differences in motion. Specifically, the mean FD was 0.52 ± 0.43 for MB scans vs. 0.40 ± 0.37 for MBME scans (*p* = 0.09), and the maximum FD was 0.096 ± 0.042 for MB scans vs. 0.101 ± 0.036 for MBME scans (*p* = 0.24) (Cohen et al., 2021b). This analysis helps ensure that any differences observed in functional connectivity metrics between the two scanning techniques are not confounded by differences in subject motion during scanning sessions. To summarize, for ME-ICA & DELMAR, there are 370–382 components left across within MBME fMRI after dropping 24 ± 6 components using ME-ICA denoising. Meanwhile, DELMAR can estimate 96, 24, and 6 components (i.e., BCNs) at the first, second, and third layers, respectively. On the other hand, for DELMAR/Denoise/Mapping, DELMAR can estimate 300, 72, 18, and 6 components at the first, second, third, and fourth layers, respectively. More details about the important hyperparameter settings and parameters of ME-ICA & DELMAR as well as DELMAR/Denoise/Mapping can be found in Supplementary Table S2. Furthermore, registering directly to MPRAGE before denoising could significantly increase resolution and processing time due to the more complex manipulations required, such as interpolation and smoothing. Therefore, denoising first reduces these complexities and potential distortions. After denoising, the images are registered to the AC-PC-aligned MPRAGE image using epi_reg provided by FSL, and subsequently, they are registered to MNI space using the anatomical transformations previously computed. Finally, the ME-ICA data were smoothed using a 4 mm FWHM Gaussian kernel and bandpass filtered with 0.01 < *f* < 0.1 Hz.

As in our previous studies (Zhang et al., 2020b; Zhang and Bao, 2022), all deep linear models were evaluated equally using well-established canonical BCNs as the ground truth templates. Following the fMRI ICA pipeline for spatially independent networks, the twelve ground truth templates (Smith et al., 2009) were used as components/spatial features. These features were employed as templates to evaluate the reconstruction performance of DELMAR. These ground truth templates derived from resting-state fMRI have been released publicly and are considered functional brain areas covering a large part of the cerebral cortex (Smith et al., 2009). The names of all ground truth templates are shown in Supplementary Table S1.

In addition, Supplementary Table S2 provides the primary hyperparameters and parameter settings of two computational frameworks to extract the hierarchical BCNs. In general, the hyperparameters include the number of layers and the number of components of each layer, i.e., the size of the layer or the size of the weight matrix/dictionary. Other important parameters are the number of iterations and the step length of gradient descent, where applicable. Since DELMAR can estimate all these hyperparameters automatically, only the maximum number of iterations and step size were given. Specifically, * indicates that this parameter needs to be set manually. Our prior research also explained determining the parameters, such as the number of iterations and step length (Zhang et al., 2020b). In fact, DELMAR is also a dimensional reduction method, so the number of deeper features, i.e., the number of BCNs, is gradually reduced. Notably, the first layer of DELMAR/Denoise is utilized to extract the BOLD signal. Thus, the validation of the two computational frameworks is the first layer of ME-ICA & DELMAR vs. second layer of DELMAR/Denoise/Mapping, the second layer of ME-ICA & DELMAR vs. second layer of DELMAR/Denoise/Mapping, and the third layer of ME-ICA & DELMAR vs. fourth layer of DELMAR/Denoise/Mapping.

### 2.5 Introduction of intensity, spatial and hausdorff metrics

In this section, we quantitatively compare the identified BCNs with the ground truth templates in three different ways. First, spatial similarity, which is largely independent of the intensity of each voxel of the identified components (Zhang et al., 2018a,b), was computed. The definition of spatial similarity is:


(10) $${\mathrm{Similarity}}_{\mathrm{Spatial}}=\frac{\left|\mathrm{Component}\cap \mathrm{Template}\right|}{\left|\mathrm{Template}\right|}$$

where $\left|\text{.}\right|$ represents binarization, which indicates the voxels above a given intensity threshold. In general, since an BCN only occupies a very small region of the entire brain area (Smith et al., 2009), the intensity threshold can be tuned by sorting all voxel intensities in descending order. In Equation 10, we utilized the top 5% as the threshold (Zhang et al., 2018a,b, 2020a,b). The spatial similarity metric measures the ratio of the intersection and the union of the identified BCN and ground truth templates (Smith et al., 2009).

In contradistinction, only considering the intensity of each voxel of the derived components, it is useful to calculate the distance between the intensities of identified BCNs and templates, i.e., ground truth templates and simulated templates.

The definition of intensity similarity is:


(11) $${\mathrm{Similarity}}_{\mathrm{Intensity}}={\left(\sum_{i=1}^{N}\frac{\left|x_{i}-y_{i}\right|}{\left|x_{i}|+|y_{i}\right|}\right)}^{-1}$$

where $\left|\text{.}\right|$ represents the absolute value. Given a threshold, the intensity similarity is calculated via summed absolute value of intensity of component (denoted as $x_{i}$ ) and template (denoted as $y_{i}$ ) divided by the absolute value of their difference. N denotes the total number of voxels. If all intensity values of the components and templates are equal, the intensity similarity approaches infinity (Zhang et al., 2020b).

Finally, to jointly consider both spatial and intensity matching, we used the Hausdorff Distance (HD) (Zhang et al., 2018a,b, 2020b):


(12) $$\begin{array}{c} X=\sum_{i=1}^{N}2\times \min\left(x_{i},y_{i}\right),\mathrm{if}\;x_{i},y_{i}\in C\cap T\\ Y=\sum_{i=1}^{M}x_{i}+y_{i},{ifx}_{i},y_{i}\in C\cup T\\ HD=\frac{X}{Y} \end{array}$$

Briefly, X represents twice the minimum intensity value of the intersection between a component and the template, while Y denotes the summed intensity value of their union. Here, C and T represent the sets of components and templates, respectively. Consequently, HD (Hausdorff Distance) simultaneously reflects intensity similarity and spatial overlap. To assess the reproducibility of MB and MBME using test–retest data, we apply HD as defined in Equation 12 to calculate the correlations of each identified component between the test and retest datasets.

## 3 Results

We employ two different computational frameworks: ME-ICA & DELMAR verses DELMAR/Denoise/Mapping to investigate the hierarchical organization of BCNs and their reproducibility from MBME fMRI. As discussed, in following sections, we hope to prove multiple hypotheses raised in the Introduction section. On the one hand, since shallow BCNs serve as a gold standard for fundamental brain functionality (Smith et al., 2009). They are widely accepted as a rigorous benchmark for evaluating novel methodologies. In this study, our primary objective is to investigate the denoising capabilities of our approach. The identification of shallow BCNs with high spatial similarity to established templates confirms that our method effectively denoises fMRI data across multiple layers. On the other hand, given that no universally accepted gold standard templates exist for meta-BCNs (Wylie et al., 2021), we emphasize their reproducibility as a key validation criterion. In particular, sections 3.1 and 3.3 provide a detailed validation of reproducible meta-BCNs identified via DELMAR.

### 3.1 Investigating the multi-layer reconstructions of BCNs and their reproducibility from MB and MBME fMRI using DELMAR

In this section, we focus on employing DELMAR to extract hierarchical BCNs from both MB fMRI and MBME fMRI of the same normal adult volunteers (Cohen et al., 2020, 2021a,b), using test–retest reproducibility metrics to validate previously proposed hypothesis that is MBME fMRI consists of more reproducible hierarchical BCNs than MB fMRI data from twelve well-known canonical BCNs (Smith et al., 2009) for the “ground truth” accuracy of the shallow-layer results from the deep linear method. In particular, ground truth templates representing critical brain functions, such as the Default Mode Network (DMN) and Auditory Network (AUD), have been identified over the past two decades through conventional computational frameworks (Smith et al., 2009) and continue to play a key role in advancing our understanding of more complex brain functionalities and validating innovative methods for fMRI analysis (Stam, 2014; Huang et al., 2018; Zhang et al., 2019, 2020a; Agarwal et al., 2023).

However, due to the lack of concrete ground truth for meta-BCNs, we employ multiple data-driven pipelines (Zhao et al., 2016; Zhang et al., 2018a,b; Satterthwaite et al., 2019; Gao et al., 2022) to generate simulated templates (sTemplates) for these meta-BCNs. Specifically, we collected twelve ground truth templates from Smith’s work (Smith et al., 2009) and applied Zhang’s method (Zhang et al., 2018a,b) to generate individual simulated fMRI signals. Next, we used DELMAR, Deep SDL (Qiao et al., 2021), and Deep ICA (Wylie et al., 2021) on each simulated fMRI signal to identify meta-BCNs, with each simulated fMRI matrix sized 100 × 906,629. Importantly, DELMAR’s hyperparameters are determined automatically, while those for Deep ICA and Deep SDL were tuned based on prior studies (Qiao et al., 2021; Wylie et al., 2021). After removing noisy BCNs and artifacts (Satterthwaite et al., 2019), we calculated spatial similarity (Zhang et al., 2018a,b) across all BCNs identified at the second layer using three computational approaches (e.g., DELMAR, Deep ICA, and Deep SDL). We then performed clustering (e.g., k-means) to categorize BCNs based on spatial similarity (Zhao et al., 2016). Finally, we created group-wise BCNs as simulated templates (sTemplates) via Gaussian smoothing (Gao et al., 2022) based on individual BCNs within each dominant cluster, covering at least 90% of subjects (Zhao et al., 2016). In conclusion, this process yielded six simulated templates for meta-BCNs.

**Figure 2:**
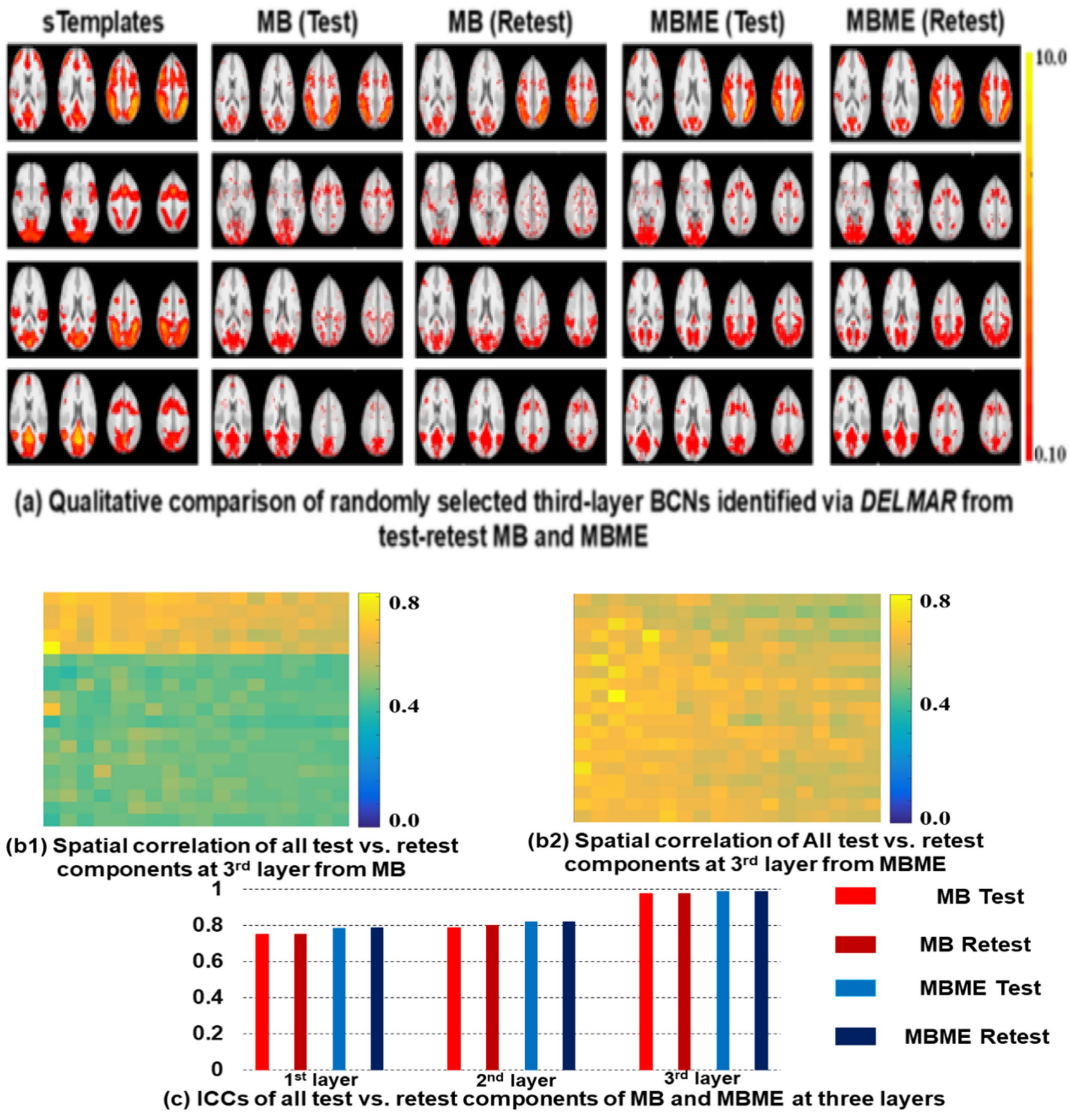
Qualitative comparison **(a)** with simulated templates in the first column (Zhang et al., 2020b), and quantitative analyses **(b,c)** identified ten 3rd layer networks via MB and MBME fMRI. The correlation matrices of test versus retest scans **(b1,b2)** demonstrate stronger reproducibility of MBME than MB at the third layer. **(c)** Shows higher ICC values of MBME than MB across all layers.

In addition, all representative slices of derived canonical BCNs from MB and MBME fMRI via DELMAR have been included in Supplementary Figure S1. In addition, the low-level Layer 1 BCNs correspond well to the ground truth templates from the simulated fMRI and this correspondence improves further for the second layer BCNs, especially for the lateralized left and right frontoparietal networks (FP-L and FP-R). The results of MBME fMRI better match the templates generated from multiple computational approaches using the simulated and real fMRI data (Zhang et al., 2020b, 2024; Zhang and Bao, 2022). Using Hausdorff metric as the spatial similarity measure to the ground truth templates, the average of the twelve Layer 1 BCNs from MBME fMRI (0.259 ± 0.039) was significantly better than for MB fMRI (0.188 ± 0.039; *p* < 10^−6^). The same was true for the Layer 2 BCNs (MBME: 0.287 ± 0.042; MB: 0.214 ± 0.030; *p* < 10^−5^). The Hausdorff similarity to the ground truth templates was better for the second layer than the first layer for both MB fMRI (*p* = 0.001) and MBME fMRI (*p* < 0.01). Furthermore, the group-wise BCNs from DELMAR of the test and retest MB and MBME fMRI data are presented for the third layer in Figure 2.

Furthermore, the BCNs at Layer 3, also considered as meta-BCN, are more complex and larger in spatial scale than those of Layers 1 and 2, as they represent the recombination of the canonical BCNs. Figure 1b1, b2 show that these high-level 3rd layer BCNs are reproducible for both MB fMRI and MBME fMRI, with better test–retest reliability than the first two layers. To evaluate the reproducibility of test vs. retest meta-BCNs derived via DELMAR from MB and MBME fMRI data, we employ the Intra-class Correlation Coefficient (ICC) (Bujang and Baharum, 2017), since ICC is considered a descriptive statistical technique suitable for quantitative measurements organized into groups. Specifically, ICC describes how strongly components in the same group resemble each other. While it is viewed as a type of correlation, unlike most other correlation measures, it operates on data structured as groups rather than paired observations. These meta-BCNs (i.e., BCNs identified at deep layers) also align with the third-layer BCNs from deep linear methods of the simulated fMRI data shown as comparisons with the templates (Zhang et al., 2018a,b, 2020b). Without the “ground truth” of meta-BCNs, we utilize the Intra-class Correlation Coefficient (ICC) as the reproducibility metric to investigate the reliability of all identified deeper BCNs.

Overall, in Figure 2c, our results demonstrate that ICC improved from 0.750 in Layer 1 to 0.789 in Layer 2 to 0.980 in Layer 3 for MB fMRI. For MBME fMRI, the results were 0.751 to 0.788 to 0.984 in the successive layers. In conclusion, the qualitative and quantitative results in this section support the hypothesis that MBME fMRI cultivates more reproducible and consistent hierarchical BCNs than MB fMRI data. Specifically, the qualitative results in Figure 2a depict multiple representative slices of four randomly selected meta-BCNs derived via DELMAR at the third layer from test vs. retest MB and MBME fMRI. In general, meta-BCNs identified from MBME showcase better reproducibility. For instance, in the first row of Figure 2, the nodes of Left Frontoparietal Network (FR-L), Dorsal Attention Network (DAN), and Salient Network (SN) are concatenated in identified meta-BCNs, but this connection is disrupted in test vs. retest MB data. Nevertheless, the connection has been successfully identified between test and retest MBME fMRI data. Additionally, in the second row of Figure 2, the visual network (at the bottom of the BCN) shows significant changes within test vs. retest MB fMRI data, while there is no significant variation throughout test vs. retest MBME fMRI data. Furthermore, in the fourth row of Figure 2, the nodes of SN (at the top of last two slices within each network) shows significant inconsistency within test vs. retest MB fMRI data, while there is no significant variation throughout test vs. retest MBME fMRI data. Meanwhile, in sub Figures 2b1,b2, it is evident that the spatial correlation of meta-BCNs derived via DELMAR at the third layer from MBME is significantly larger than that from MB. Moreover, ICC values across all hierarchical BCNs of all subjects further demonstrate that various BCNs revealed from MBME fMRI are more reproducible and consistent than those from MB fMRI.

Notably, in Figure 2a, the values (ranging from red to yellow), e.g., intensities, within each BCN reflect the activation intensity of different brain regions, with higher values indicating stronger activation (Agarwal et al., 2023). This information is essential for understanding neural activity levels using fMRI (Dimova et al., 2024; Oathes et al., 2021; Wang et al., 2021). For instance, in Figure 2a, meta-BCNs at the first row showcase a stronger activation (in yellow color) than other meta-BCNs.

To further mitigate demographic sensitivity and inherent variability in the identified BCNs, we have utilized a methodology introduced by Agcaoglu et al. (2015) designed to balance variability with reproducibility effectively. This approach involves generating group-wise BCNs by averaging multiple summed BCNs that exhibit the highest similarity to either a single ground truth template (for shallow-layer BCNs) or a simulated template (for deep-layer BCNs), as identified by various computational frameworks across all subjects. Notably, these group-wise BCNs not only represent the functional structure but also achieve an effective balance between demographic sensitivity and inherent variability, as detailed in Agcaoglu et al. (2015).

Besides, the qualitative results of other meta-BCNs identified via DELMAR at the third layer and the quantitative correlations of all hierarchical BCNs revealed via DELMAR at the first and second layers can be viewed in Supplementary Figures S2, S3. Specifically, in Supplementary Figure S2, the qualitative results in the first, second, and third rows identified from test vs. retest MB showcase significant variance between test and retest, while no significant changes are observed within meta-BCNs from test vs. retest MBME. Other meta-BCNs in the fourth, fifth, and sixth rows revealed from MB are disrupted in the Left Frontoparietal Network (FP-L), Sensory Motor (SM), and SN. In Supplementary Figure S3, the quantitative correlations indicate that the spatial reproducibility of BCNs from MBME is stronger than those from MB.

### 3.2 Investigating lower-level reconstructions of BCNs via integrated deep linear denoising method and their reproducibility from MBME fMRI

**Figure 3:**
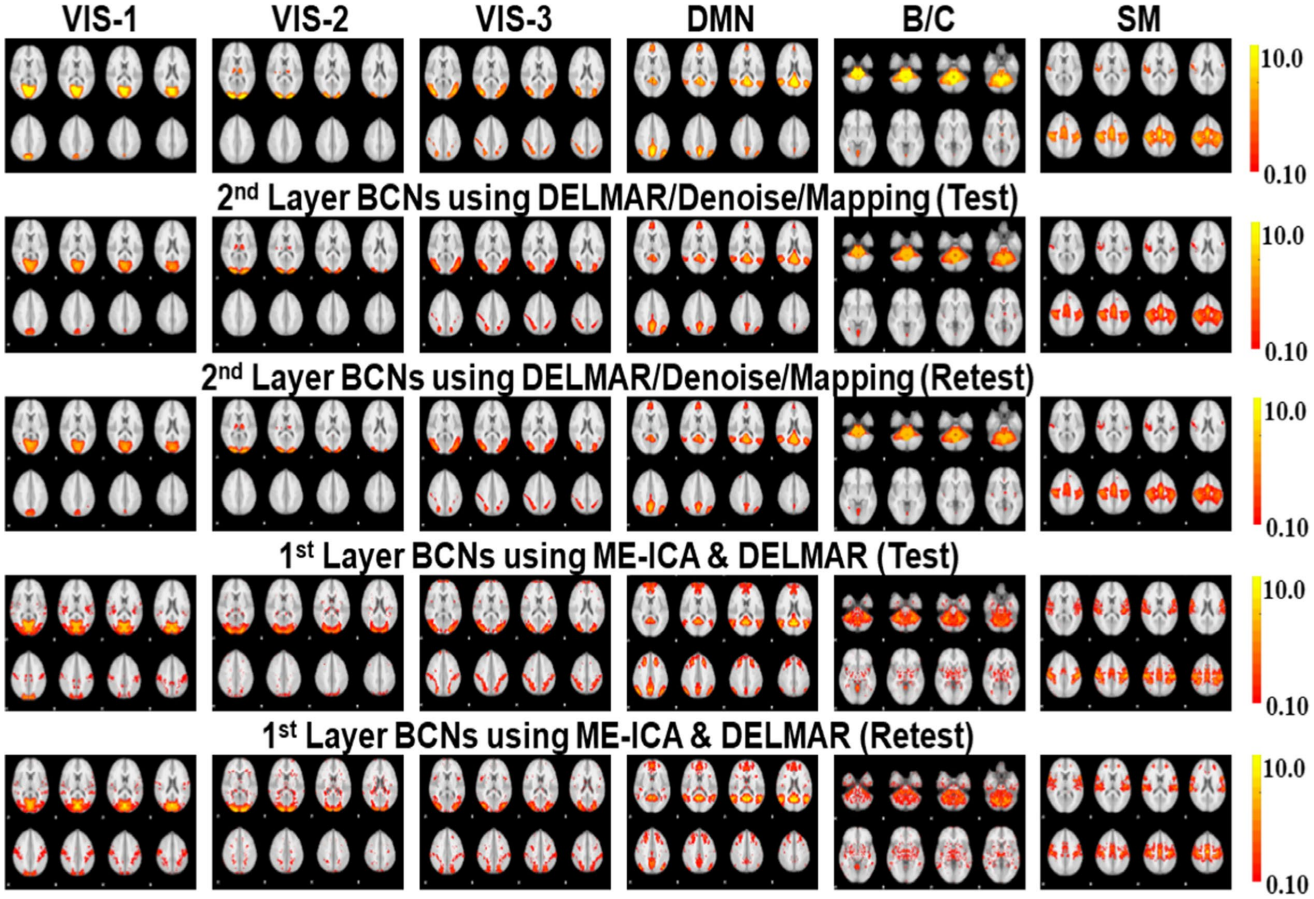
Comparison of six 1st and 2nd layer networks via two computational frameworks: DELMAR with ME-ICA Denoising verses DELMAR with 1st Layer Denoising. Ground truth templates are in the top row (please refer to Supplementary Table S1 for detailed information of networks #1-#6 and their abbreviations).

**Figure 4:**
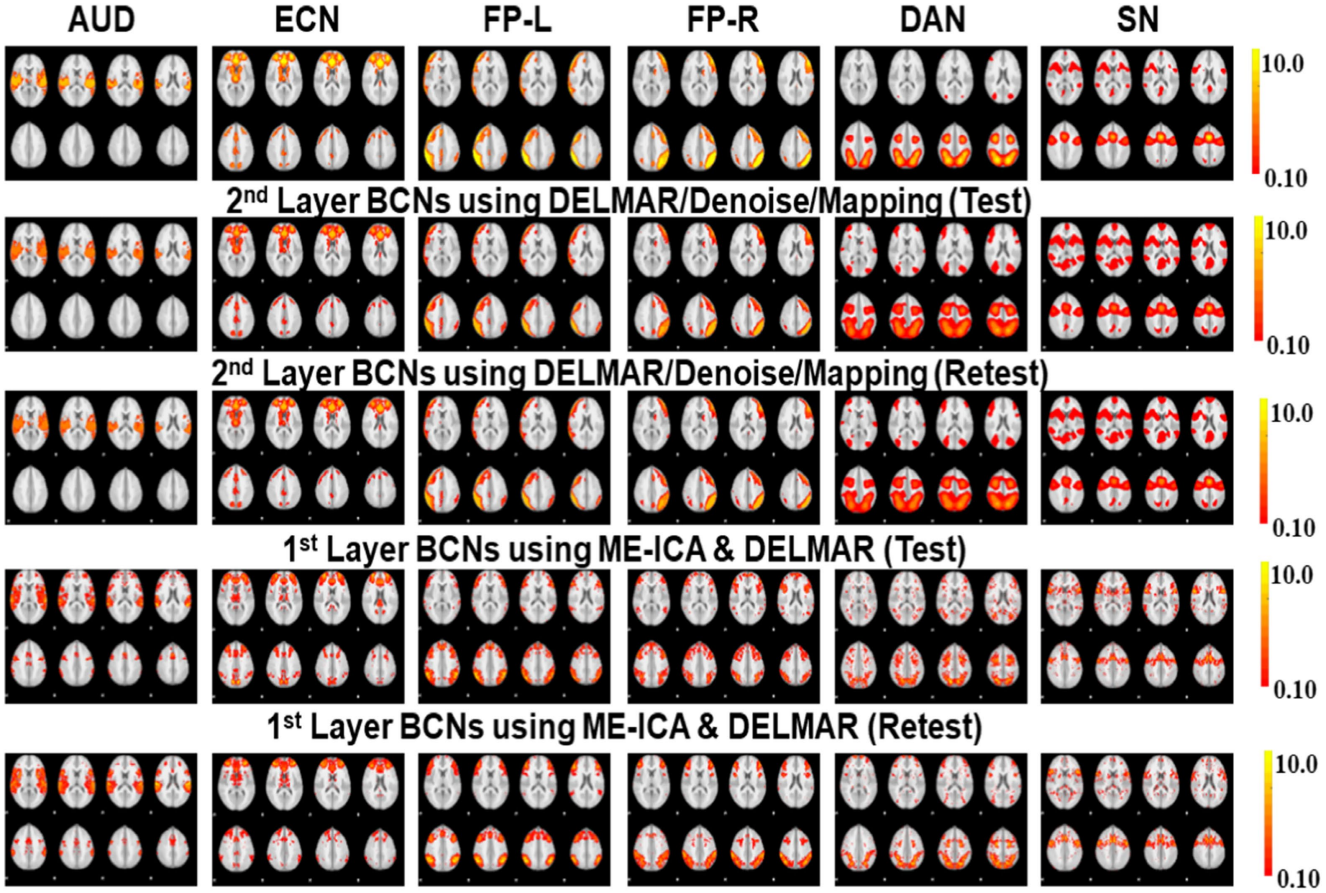
Comparison of another six 1st and 2nd layer BCNs via two computational frameworks, with ground truth templates in the top row (please refer to Supplementary Table S1 for details of network #7-#12 and their abbreviations).

**Figure 5:**
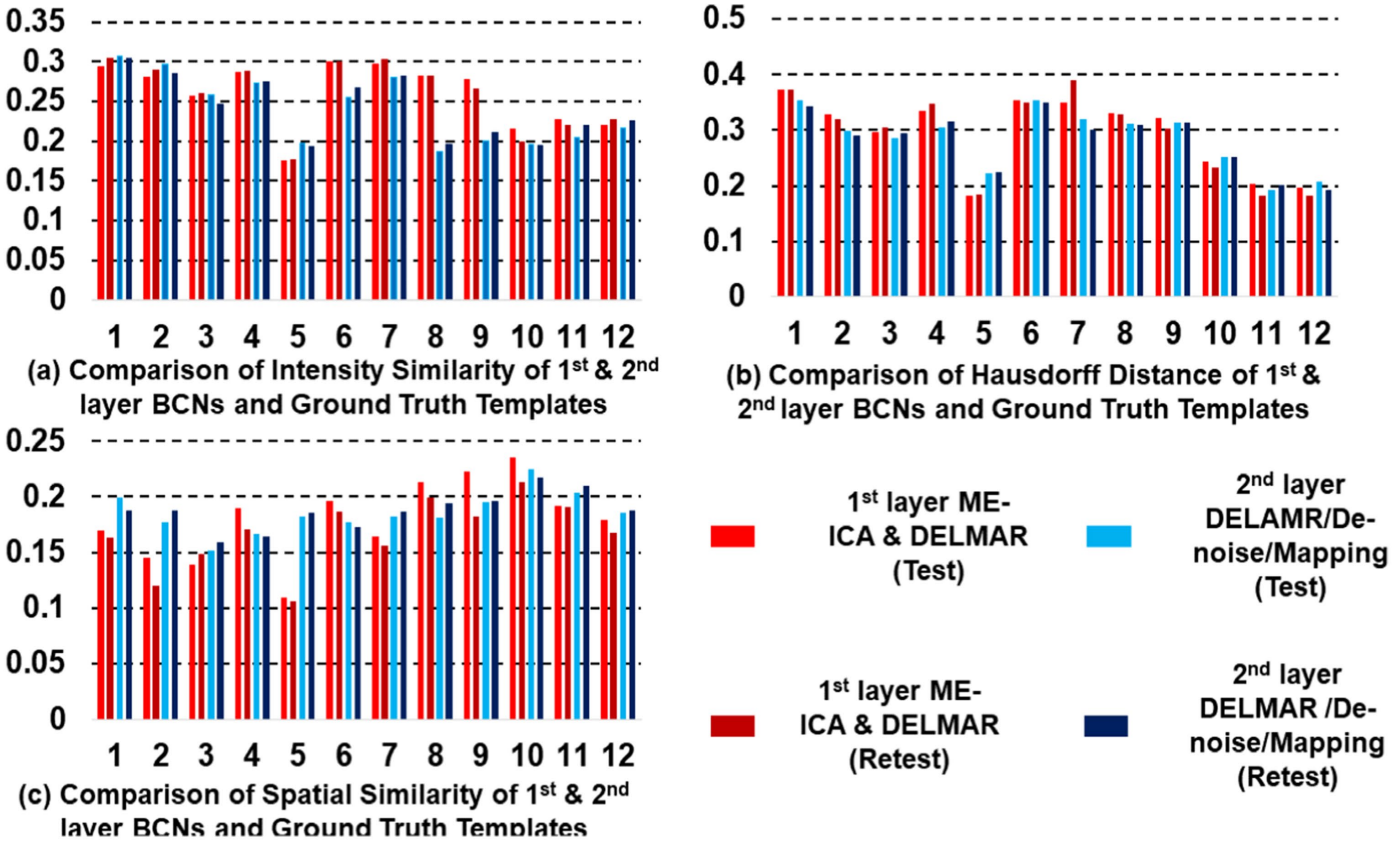
Quantitative comparisons of the twelve first- and second-layer BCNs identified by two distinctive frameworks for **(a)** intensity similarity to the ground truth templates; **(b)** the Hausdorff Distance to the ground truth templates that jointly considers intensity and spatial similarity; and **(c)** spatial similarity to the ground truth templates; the test–retest results obtained by ME-ICA & DELMAR is in light and dark green, respectively; similarly, the test–retest comparison provided by DELMAR/Denoise/Mapping is in light and dark purple.

In this section, we plan to prove another hypothesis is that DELMAR/Denoise/Mapping cultivates more reproducible results than ME-ICA & DELMAR in both lower-level and higher-level BCNs. The hypothesis aims to validate the performance of DELMAR/Denoise/Mapping superior to ME-ICA & DELMAR. Naturally, proving the proposed hypothesis can also validate the advances of DELMAR. The following spatial results (Figures 3–5) are a group-wise qualitative presentation and quantitative analysis of shallow features, i.e., low-level BCNs. Note that the first layer BCNs after ME-ICA denoising are equivalent to the second layer BCNs of the integrated DELMAR framework since its first layer is used for BOLD denoising like that of ME-ICA (please refer to Figure 1).

The results show that ME-ICA & DELMAR and DELMAR/Denoise/Mapping both yield BCNs that are similar to the ground truth templates, whereas DELMAR/Denoise/Mapping yields relatively larger intensities that better match the ground truth templates (Figures 3, 4). On the contrary, there are more noisy areas in ME-ICA & DELMAR, e.g., the fourth and fifth column in Figure 1, compared with DELMAR/Denoise/Mapping. For instance, although DAN and SN identified via ME-ICA & DELMAR (shown in the fourth and fifth rows in Figure 4) are comparable to the ground truth templates, most activated areas could be disrupted, compared to the results provided by DELMAR/Denoise/Mapping.

Hence, BCNs from DELMAR/Denoise/Mapping have better spatial similarity to the ground truth templates than those from ME-ICA & DELMAR. This illustrates the trade-off between intensity and spatial matching due to the larger norms of their iterative operators and sparse operators instead of ICA (Zhang et al., 2020b, 2024; Zhang and Bao, 2022).

The quantitative comparisons across the two different frameworks for intensity similarity, spatial similarity, and the Hausdorff distance are shown in Figure 5. These quantitative comparisons clearly demonstrate that DELMAR/Denoise/Mapping provides adequate intensity matching (please refer to Figure 5a) since their convergence velocity is relatively slow (Zhang et al., 2020b). Therefore, DELMAR/Denoise/Mapping can reconstruct the most accurate connectivity strengths of each component from input fMRI signals, consistent with the theory proved in our previous study (Zhang et al., 2020b). Moreover, considering spatial similarity, the noise intensity is reduced rapidly across iterations due to the sparse operator included in DELMAR. Thus, due to the noise having smaller intensity than the signal, it is reduced gradually via the sparsity operator, which benefits accounting for DELMAR, yielding the best spatial similarity results for most networks (please refer to Figure 5c). This result is also predicted by theoretical analyses by Zhang et al. (2020b).

**Figure 6:**
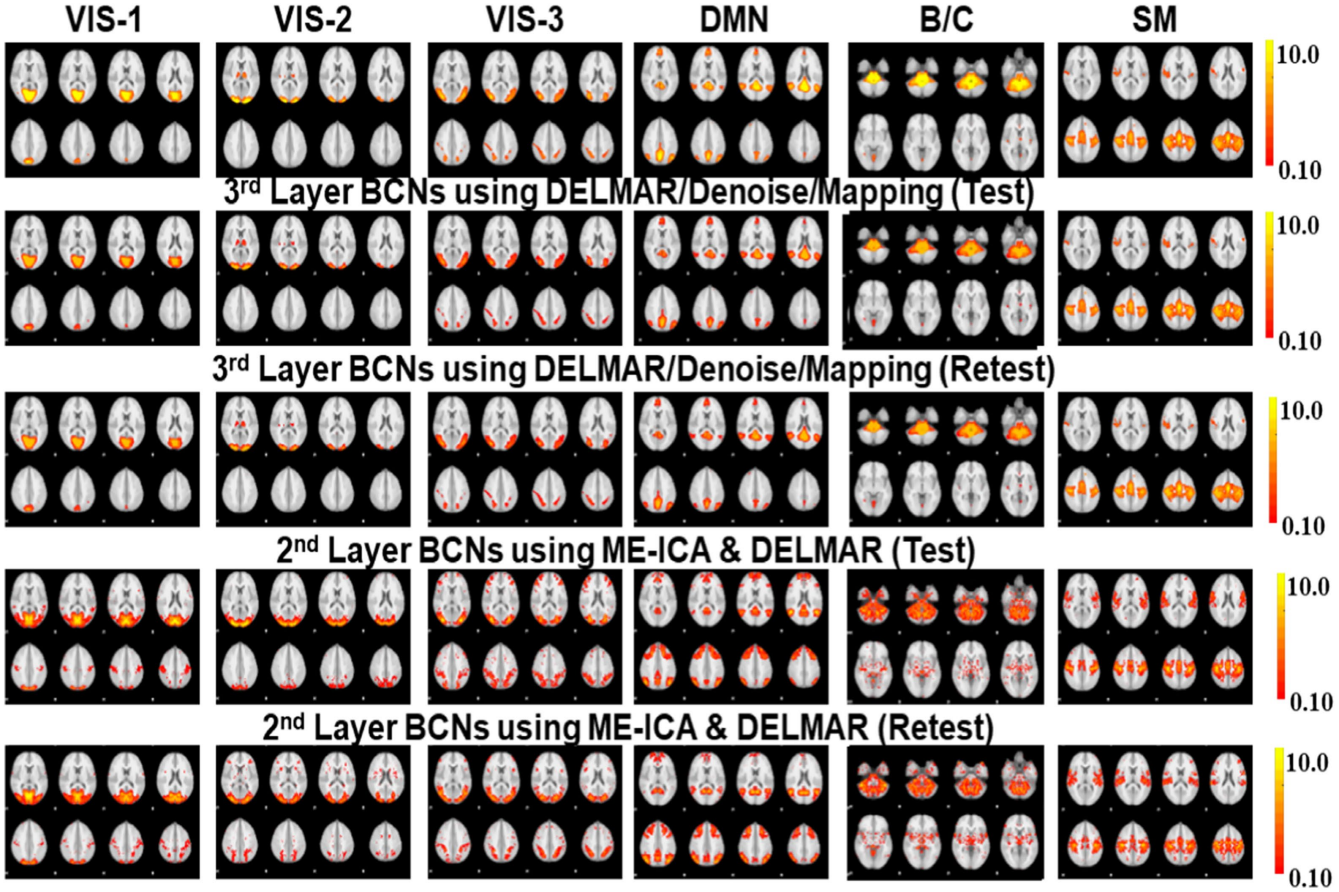
Comparisons of six BCNs, i.e., #1-#6, derived from the second and third layer BCNs of ME-ICA & DELMAR and DELMAR/Denoise/Mapping, respectively. Each column includes one test–retest representative second- or third-layer networks via two computational frameworks, matched across models in each row, with the ground truth templates in the top row (please refer to Supplementary Table S1 for details of network #1-#6 and their abbreviations).

**Figure 7:**
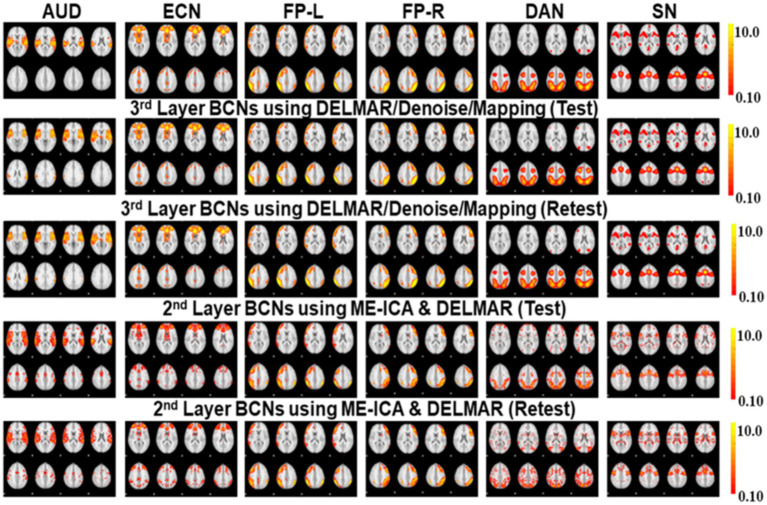
Comparisons of another six BCNs, i.e., #7-#12, derived from the second and third layer BCNs of ME-ICA & DELMAR and DELMAR/Denoise/Mapping, separately. Each column includes one test–retest representative second or third layer BCNs via two computational frameworks, matched across models in each row, with the ground truth templates in the top row (please refer to Supplementary Table S1 for details of network #7-#12 and their abbreviations).

**Figure 8:**
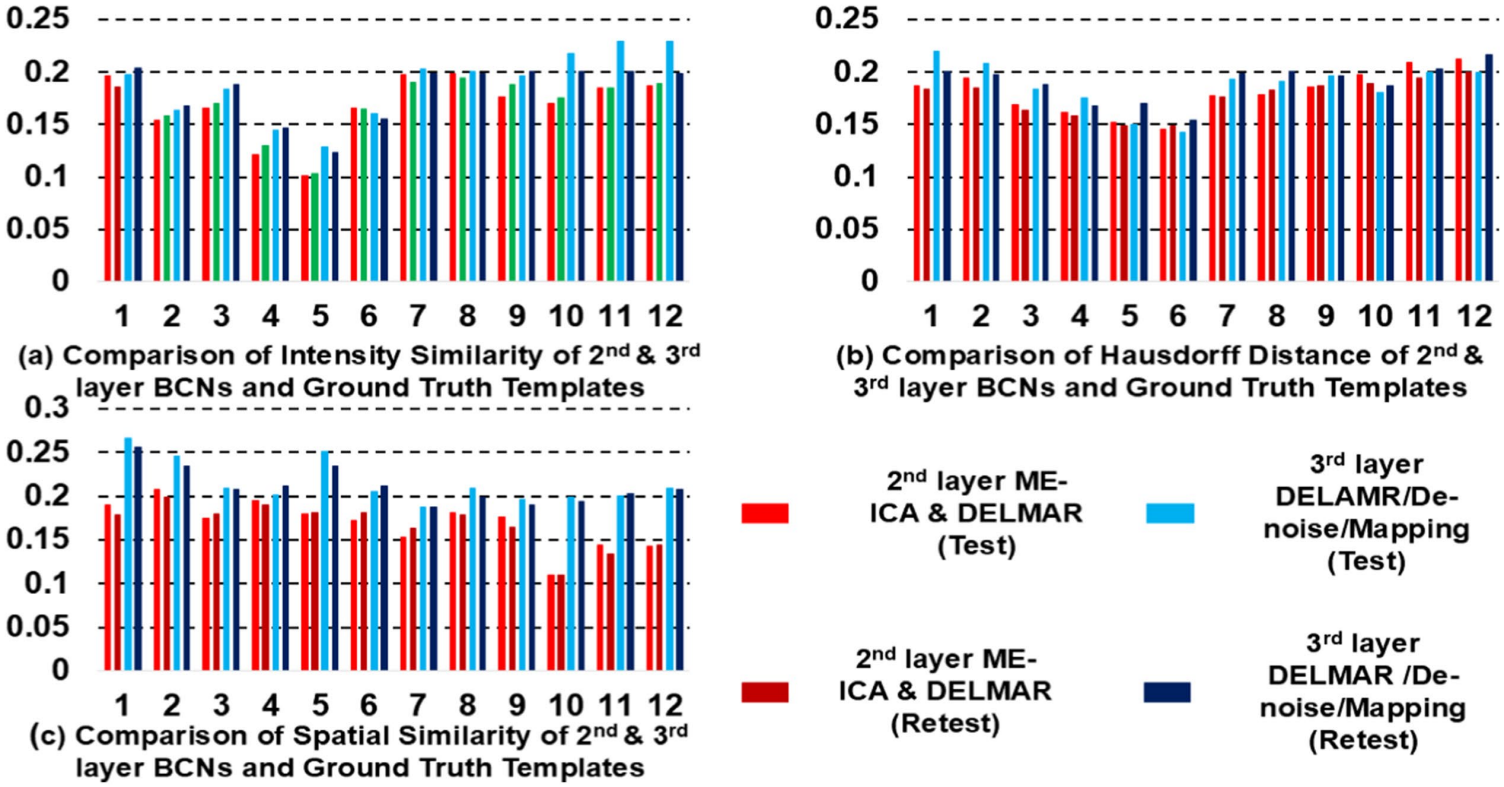
Quantitative comparisons of the twelve second- and third-layer BCNs identified by two distinctive frameworks for **(a)** intensity similarity to the ground truth templates; **(b)** the Hausdorff Distance to the ground truth templates that jointly considers intensity and spatial similarity; and **(c)** spatial similarity to the ground truth templates. The test results obtained by ME-ICA & DELMAR and DELMAR/Denoise/Mapping are in light red and blue, respectively; meanwhile, the retest comparisons are in dark red and blue.

Moreover, all proposed frameworks can be evaluated by Hausdorff Distance to investigate both intensity and spatial similarity (please refer to Figure 5b). There was no significant difference between ME-ICA & DELMAR and DELMAR/Denoise/Mapping in most BCNs. Similarly, the sparsity operator of DELMAR helps them outperform ICA. In summary, based on qualitative and quantitative comparisons, the test–retest results demonstrate that first and second-layer BCNs extracted by ME-ICA & DELMAR and DELMAR/Denoise/Mapping are both reproducible. Next, the deeper layer BCNs were investigated. Figures 6–8 provide the second- and third-layer results of ME-ICA & DELMAR and DELMAR/Denoise/Mapping, with qualitative and quantitative comparisons.

In addition, we compare DELMAR/Denoising/Mapping with ME-ICA/DELMAR using Hausdorff distance as the similarity measure to the ground truth templates. In detail, the averaged similarity and standard deviation of the twelve Layer 2 BCNs derived via DELMAR/Denoising/Mapping versus Layer 1 BCNs derived via ME-ICA & DELMAR is 0.184 vs. 0.167, 0.030 vs. 0.028, respectively. The averaged similarity of the twelve Layer 3 BCNs derived via DELMAR/Denoising/Mapping versus Layer 2 BCNs derived via ME-ICA & DELMAR is 0.184 vs. 0.167, 0.030 vs. 0.028, respectively. Similarly, the DELMAR/Denoise/Mapping is continuously denoising in the deep layers, e.g., the third layer, and the results are not significantly varied, based on the t-test.

**Figure 9:**
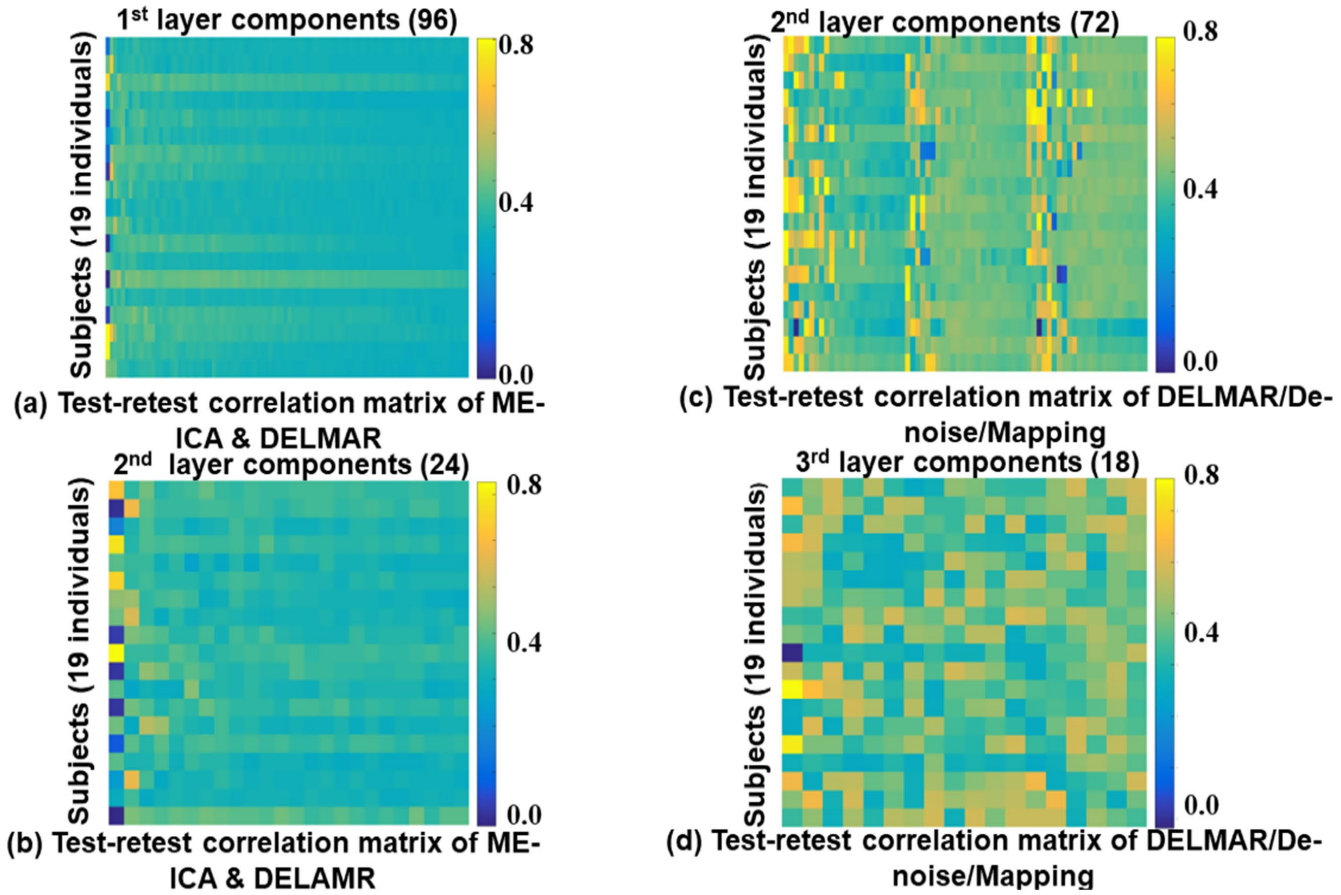
Test–retest similarity comparisons of BCNs from first, second layer of ME-ICA & DELMAR and second, third layer of DELMAR/Denoise/Mapping. Each element represents the spatial similarity of the identified component from test–retest resting-state MBME fMRI data. In detail, **(a,b)** present the test–retest comparisons of extracted first and second layer BCNs using ME-ICA & DELMAR; **(c,d)** provide a similar comparison of revealed results using DELMAR/Denoise/Mapping.

Furthermore, all second and third-layer BCNs identified via two computational frameworks still strongly correlate with the twelve ground truth templates (Smith et al., 2009). By iteration using the sparsity operator, the intensity of noise components is gradually reduced. Thus, the Hausdorff Distance of most third-layer BCNs extracted by DELMAR/Denoise/Mapping versus twelve ground truth templates is increased (please refer to Figure 9c). The reproducibility of BCNs, e.g., first- and second-layer BCNs of ME-ICA & DELMAR, second and third layer BCNs of DELMAR/Denoise/Mapping are investigated in the following sections.

In Figure 9, four test–retest correlation matrices demonstrate that the reproducibility of DELMAR/Denoise/Mapping is better than ME-ICA & DELMAR. It is easy to observe that most of the identified components via DELMAR/Denoise/Mapping have a larger correlation, e.g., some correlations approach a very high value of 0.80. Moreover, although the correlation values of DELMAR/Denoise/Mapping are relatively reduced in deeper layers, most of the test–retest correlations are still more extensive than the results obtained by ME-ICA & DELMAR. Considering the performance of DELMAR/Denoise/Mapping, the extracted top components shown in (c) have the largest correlation due to the similar performance of low-rank decomposition (Wen et al., 2012; Shen et al., 2014). In summary, these correlations demonstrate that the results of both computational frameworks are reproducible and that those of MBME fMRI are better than MB fMRI.

Based on these preliminary qualitative and quantitative comparisons, it is clear that these two computational frameworks can successfully extract first- and second-layer BCNs that are very similar to ground truth templates from ICA (Smith et al., 2009). It also indicates that the shallow (lower level) organization of MBME fMRI includes these canonical BCNs (Smith et al., 2009). Furthermore, compared to ME-ICA&DELMAR, DELMAR/Denoise/Mapping produces less noisy components that are more spatially similar to the ground truth templates, as shown by the bars included in Figure 5c. Interestingly, as shown in Figures 3, 4, 6, 7, some BCNs can be identified across multiple layers. This phenomenon suggests that BCNs identified at deeper layers typically exhibit higher spatial similarity with the templates and/or improved reproducibility, underscoring the capability of deep learning frameworks to detect consistent BCNs and effectively denoise them at deep levels (Zhang et al., 2019, 2020a). Additionally, this observation may also indicate that certain BCNs are involved in deeper and complex brain functionality (Kaefer et al., 2022).

### 3.3 Investigating the highest-level reconstructions of BCNs via integrated deep linear denoising method and their reproducibility from MBME fMRI

**Figure 10:**
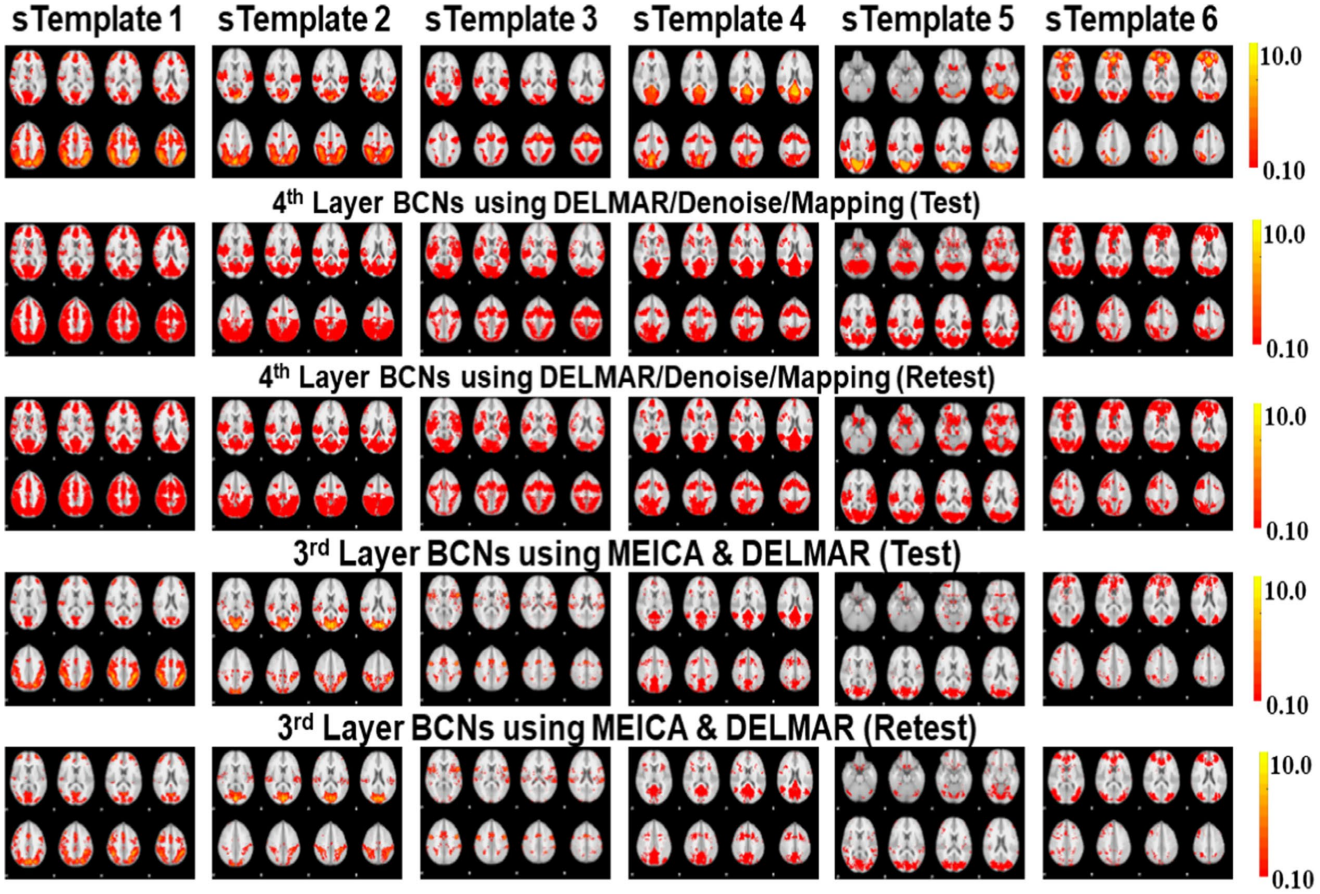
Test–retest comparisons of six extracted 3rd and 4th layer BCNs using ME-ICA & DELMAR and DELMAR/Denoise/Mapping. The first row provides eight representative slices of simulated templates. The second and third row present the test–retest corresponding BCNs identified by DELMAR/Denoise/Mapping; the fourth and fifth rows provide similar results of ME-ICA & DELMAR.

Based on previous results, we explore high-level BCNs, e.g., meta-BCNs, extracted in the third and fourth layers, recombine shallow BCNs. In recent work, these high-level BCNs have been named ‘meta-networks’ (Wylie et al., 2021). It means that a single BCN usually contains entire/partial nodes of other shallow BCNs, i.e., the spatially independent BCNs introduced by early resting-state fMRI research (Smith et al., 2009). For example, some BCNs extracted via DELMAR/Denoise/Mapping as the deeper features include the nodes of Executive Control Network (ECN), DAN, SN, FP-L, Right Frontoparietal (FP-R), and SM (Smith et al., 2009), which therefore appears to be a spatially “global” network, whereas ME-ICA & DELMAR recombines the partial nodes of FP-L, FP-R, and DMN (Smith et al., 2009), e.g., partial areas of precuneus. In particular, Figure 10 presents all extracted and reproducible high-level BCNs. In the top row, we provide the eight representative slices of high-level BCNs revealed via previous simulated experiments (Zhang et al., 2020b). For every two adjacent rows, six extracted BCNs are based on test–retest MBME fMRI datasets. In general, all BCNs extracted via the two computational frameworks are reproducible. Nevertheless, the results of DELMAR/Denoise/Mapping contain more areas than ME-ICA & DELMAR.

Overall, a single BCN extracted by DELMAR/Denoise/Mapping includes three or more entire/partial areas of several shallow BCNs, whereas some BCNs extracted by ME-ICA&DELMAR contain fewer functional nodes/areas of shallow BCNs. There are even some disrupted functional areas due to the independence constraints of ICA applied on the shallow layer that may influence the performance of DELMAR in deeper layers. Our previous experimental and theoretical analyses have shown that ICA cannot easily recombine the overlapping shallow features due to its spatiotemporal independent constraints (Zhang et al., 2019, 2020a,b).

Additionally, DELMAR/Denoise/Mapping relatively weakly recombines the previous layer’s features into its deeper layer. In contrast, the first and fourth columns of ME-ICA & DELMAR in the second layer (please refer to the first and fourth columns in Figure 10) overlap with partial areas of the Visual Network #3 (VIS-3) or FP-L, FP-R, respectively. The second layer BCNs of DELMAR/Denoise/Mapping show increased spatial similarity (please refer to Figure 9c), highlighting the dramatic improvement in spatial similarity. More importantly, we present all high-level BCNs with high reproducibility (e.g., the averaged test vs. retest Hausdorff metric of each high-level BCN identified via ME-ICA & DELMAR and DELMAR/Denoise/Mapping across all subjects is larger than 0.40).

In detail, Figure 10 presents the most representative slices of each identified high-level BCN via DELMAR/Denoise/Mapping. Since the similarity and reproducibility of BCNs derived by DELMAR/Denoise/Mapping are better than ME-ICA & DELMAR, we concentrate on the organization of these BCNs. The first BCN, shown in the first column of Figure 10, can be considered the ‘global network’ since it contains the nodes of the Visual Network #1 (VIS-1), ECN, including the insulae, pre-supplementary motor areas (pre-SMA), premotor areas, and most areas of FP-L and FP-R, partial areas of DAN and SN, and even partial precuneus area of DMN. The second BCN includes most areas of AUD and DAN and major areas of VIS-1, and VIS-3, containing partial areas of the occipital lobe and some nodes of DMN, Brain Stem/Cerebellum (B/C), FP-L, FP-R, and DAN. For the third BCN, it occupies a very large area of Auditory Network (AUD), most nodes of SN, and a partial area of VIS-2. The fourth high-level BCN is dominated by DMN since it almost contains the entire precuneus area. Meanwhile, most areas of VIS-1 and partial nodes of DAN and SN are involved. The fifth and sixth BCNs are dominated by visual areas, such as VIS-1 and VIS-3. The major difference is that the fifth BCN occupies most areas of AUD which is considered a combination of visual and auditory functions. Moreover, some nodes of SM are involved. Furthermore, the sixth BCN can be differentiated by involved nodes of ECN, FP-L, and B/C.

Recently, two spatial maps of high-level networks notably differed from previously reported ICA networks, consistent with hypothesized high-level networks (Wylie et al., 2021). One high-level network encompasses both the occipital lobe visual system as well as frontoparietal association regions and correlates strongly with VIS-1, partial VIS-3, and DAN.

The second high-level network, derived by lower-order ICA, basically encompassed dorsolateral prefrontal and parietal control regions, strongly matching the ground truth templates (Smith et al., 2009; Wylie et al., 2021). Notably, however, the correlation between this second high-level network and the DMN template was very weak. In the Wylie et al. (2021) study, these meta-correlations indicate that this BCN is more accurately considered an BCN partially encompassing the DMN. Interestingly, at the highest levels of the connectivity dendrogram, Wylie et al. (2021) reported that the hierarchical BCNs initially separate the brain into Visual/Attention and Default/Control networks. However, unlike hierarchical clustering analyses, ICA spatial maps were not always neatly subdivided into nested subnetworks as independent component numbers increased (Wylie et al., 2021). Furthermore, these two identified high-level networks are qualitatively similar to high-level BCNs #1 and #2.

**Figure 11:**
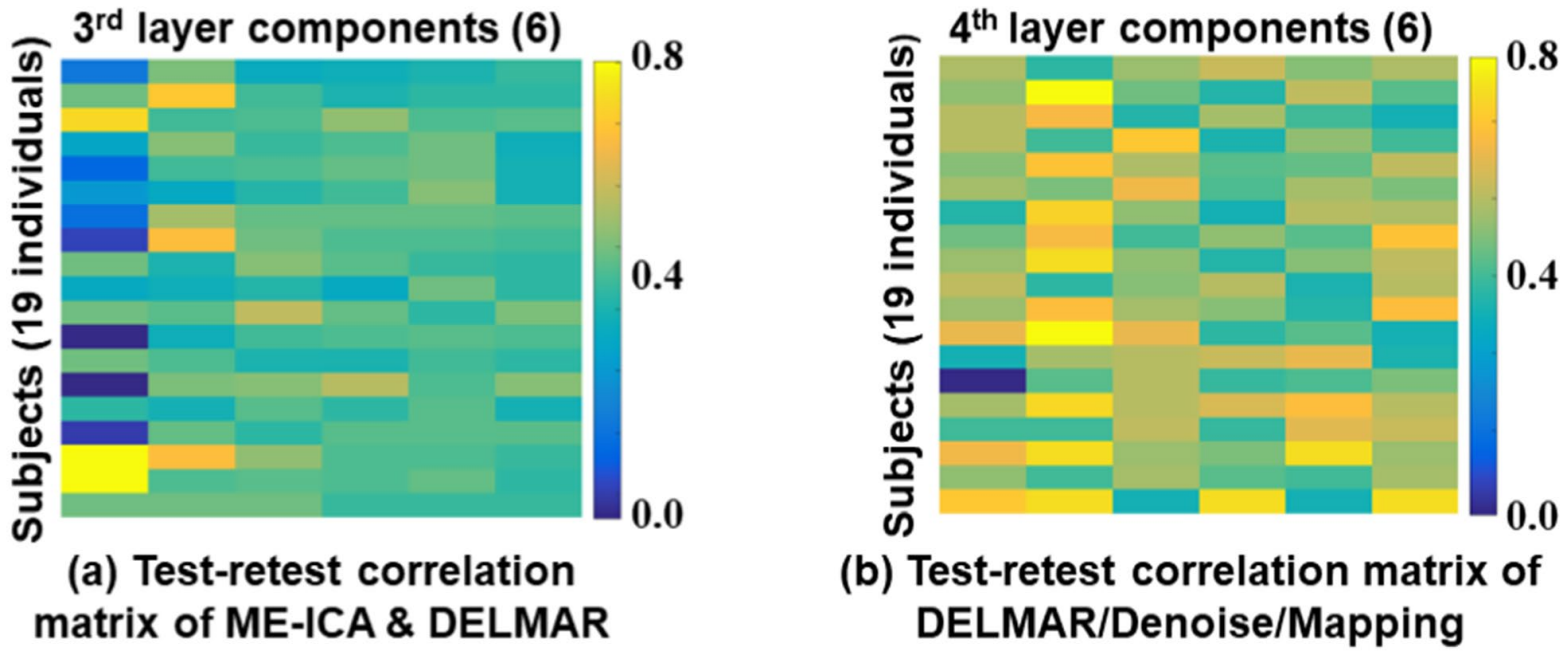
Test-retest correlations of high-level BCNs, i.e., meta-BCNs, derived by two computational frameworks [ME-ICA & DELMAR in subfigure **(a)** and DELMAR/Denoise/Mapping in subfigure **(b)**] across all individuals using Hausdorff Metric (Zhang et al., 2018a,b, 2020b).

Furthermore, the quantitative examinations of the reproducibility of these two computational frameworks are calculated and visualized in Figure 11. There are six meta-BCNs extracted via the two frameworks based on test–retest MBME fMRI data, and we calculate the correlations of all components. The correlation matrices are 19 × 6. Comparing Figures 9a,b, DELMAR/Denoise/Mapping shows better reproducibility of the meta-BCNs than ME-ICA & DELMAR for all 19 subjects. Additionally, Figure 11c compares the extracted high-level BCNs with simulated templates (refer to the first row in Figure 10). DELMAR/Denoise/Mapping demonstrates better similarity than ME-ICA & DELMAR for every extracted BCN. Meanwhile, the correlation of meta-BCNs identified via DELMAR from MBME with the ground truth templates (Smith et al., 2009) is also included in Supplementary Figure S4.

Besides, to further showcase the efficiency of DELMAR, we compare the post-processing time (i.e., the time required to separate all hierarchical BCNs via DELMAR) using both ME-ICA & DELMAR and DELMAR/Denoise/Mapping to reveal hierarchical BCNs from MBME fMRI data. The time consumption (averaged time ± standard deviation) for ME-ICA & DELMAR across all subjects is 2901.65 ± 196.96 s, while DELMAR/Denoise/Mapping requires 2651.65 ± 76.96 s. These results further demonstrate the efficiency of the DELMAR/Denoise/Mapping framework in investigating hierarchical BCNs from MBME fMRI data.

**Figure 12:**
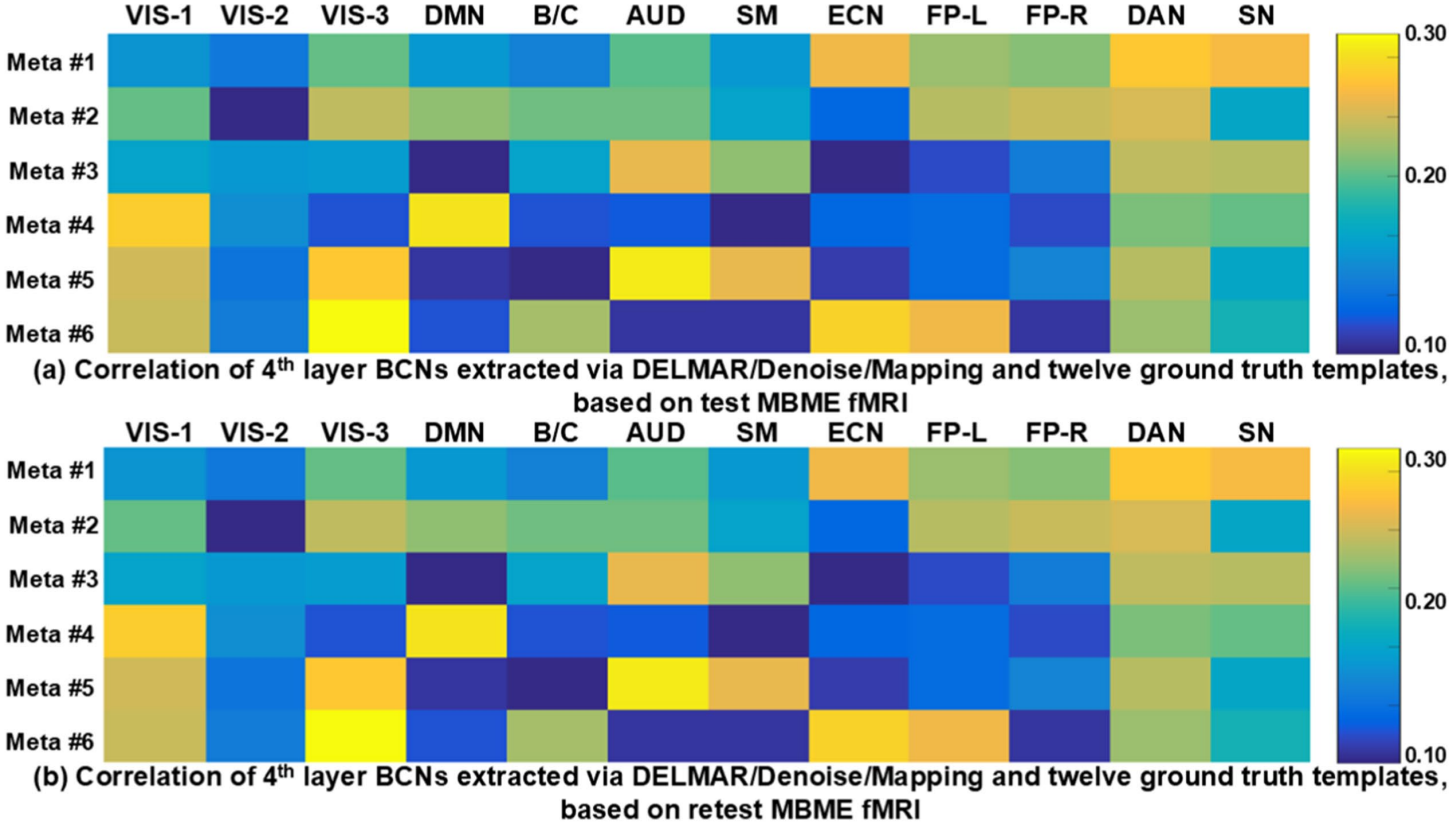
This figure illustrates the correlations between six test-retest meta-BCNs in subfigures **(a)** and **(b)**, respectively, i.e., high-level BCN, derived by DELMAR/Denoise/Mapping and twelve ground truth templates (Smith et al., 2009). The abbreviations of each ground truth template can refer to Supplementary Table S1.

Lastly, to clearly depict the correlations between the six reproducible meta-BCNs derived via DELMAR and the twelve canonical BCNs (Smith et al., 2009), we present Figure 12. This figure illustrates the connections between each meta-BCN and the canonical BCNs, with correlations calculated using the Hausdorff metric. Notably, yellow and green indicate strong correlations, ranging from 0.20 to 0.30, while blue represents weaker connections, with values around 0.10. Notably, each meta-BCN integrates three or four canonical BCNs. For instance, meta-BCN #1 combines multiple canonical BCNs, including VIS-3, AUD, ECN, FP-L, FP-R, DAN, and SN, resulting in a “global meta-BCN.” Similarly, meta-BCN #2 connects VIS-1, VIS-3, DMN, AUD, FP-L, FP-R, and DAN. Meta-BCNs #4, #5, and #6 also show strong connections with VIS-1, although only meta-BCN #4 concurrently integrates VIS-1 and DMN. Throughout Figure 12, DAN is frequently active across most meta-BCNs, likely due to its role in enabling the brain to focus on various external information sources, such as visual, auditory, olfactory, and somatosensory inputs (Markett et al., 2022). In contrast, VIS-2 is rarely integrated with other BCNs, possibly due to its specific functional role, such as extracting the shape, size, position, and number of objects (Shoham et al., 2024). Additionally, the correlations between test–retest meta-BCNs and the twelve canonical BCNs indicate no significant differences, underscoring the strong reproducibility of these six meta-BCNs. More importantly, our recent work (Zhang et al., 2024) demonstrated that meta-BCNs remain highly reproducible even with a larger augmented dataset, enhancing the robustness of our approach. These comparable results validate the effectiveness of our method across various sample sizes.

## 4 Discussion

We have introduced two computational frameworks to extract reproducible hierarchical spatial features in MBME fMRI data. These frameworks bridge the gap between traditional shallow linear models (Andersen et al., 1999; Beckmann and Smith, 2005; Calhoun et al., 2001; Hyvarinen, 1999; Lee et al., 2011; Lee et al., 2016; Mairal et al., 2010; Mckeown and Sejnowski, 1998) and newer deep neural networks (DNNs) (Hu et al., 2018; Huang et al., 2018; Dong et al., 2020; Zhang et al., 2020a).

The main advantages of the proposed algorithms are their ability to efficiently map the hierarchical organization of BCNs without requiring large amounts of fMRI data or high-performance computing clusters with GPUs or TPUs. DELMAR is also more explainable than DCAE and DBN, as we have shown through theoretical predictions of their relative performance in our previous investigations, which are validated via comparisons with peer methods (Zhang et al., 2020b). Furthermore, convergence to a unique fixed point can be guaranteed for DELMAR with alternative convex optimization functions, unlike DNNs, where such convergence is rarely achieved in practice, especially at the individual level. This is crucial given the recent realization that real-world imaging applications often suffer from underspecification, resulting in wildly unpredictable performance from any particular DNN due to convergence to different local optima from different random initial conditions despite identical training data and hyperparameters (D’Amour et al., 2020). In particular, DELMAR employs ADMM, an alternative optimization algorithm particularly well suited to optimize convex/alternative convex problems (Jain et al., 2013; Nishihara et al., 2015). It also utilizes the Rank Reduction Operator (RRO) for data-driven determination of all hyperparameters, which can be considered an intelligent factorization method. This is a major advantage over many conventional shallow data-driven fMRI connectivity reconstruction methods and other peer methods, as well as more complex deep nonlinear models, all of which must be manually tuned for hyperparameter settings. Thus, as predicted, DELMAR/Denoise/Mapping will benefit real-world imaging applications, such as fMRI. Naturally, the DELMAR/Denoise/Mapping approach is capable of outperforming other conventional methods, e.g., hierarchical clustering, focused on revealing hierarchical BCNs. For instance, hierarchical clustering would be unlikely to integrate two BCNs without spatial similarity into a meta-BCN. However, several studies (Vossel et al., 2014; Chand et al., 2017; Zhang et al., 2018a,b; Zhang et al., 2024) have demonstrated that multiple canonical BCNs with lower spatial similarity (e.g., the DMN and SN) can indeed be integrated into a single meta-BCN at the deep layers of a computational framework.

In this research, we not only validate the proposed computational framework named DELMAR/Denoise/Mapping but also successfully identify six reproducible fourth-layer BCNs, i.e., high-level networks. Both the shallow and deep BCNs demonstrate the superiority of MBME fMRI over MB fMRI, attributable to the former’s better specificity for the BOLD fMRI signal. By evaluating the first layer reconstructions of two computational frameworks, we find that ME-ICA & DELMAR and DELMAR/Denoise/Mapping can both accurately reconstruct conventional BCNs using MBME fMRI, whereas the reconstruction is not as accurate using MB fMRI. These results can be explained by the unique mix of mathematical operators used in our prior work (Zhang et al., 2020b). Overall, DELMAR/Denoise/Mapping provided reproducible and accurate reconstructions assessed via intensity matching, spatial matching, and Hausdorff Distance, compared with ME-ICA & DELMAR in the first and second layers. This could be attributed to its joint use of sparsity and rank reduction operators in conjunction with the ADMM optimizer. Furthermore, in Figure 10, we discovered that deeper features, such as the third layer BCNs, are recombination of the shallow layer networks and have the best similarity with simulated templates. For example, in Figure 11, all similarity measures for DELMAR/Denoise/Mapping are higher than for ME-ICA & DELMAR. This further validates the superiority of DELMAR/Denoise/Mapping in the deeper layers.

Moreover, ME-ICA & DELMAR produce relatively weak reproducible components in the third layer compared with the equivalent BCNs identified via DELMAR/Denoise/Mapping at its fourth layer. This can be attributed to ICA’s relatively fast convergence rate and independence constraint (Zhang et al., 2020b). The mathematical evaluation framework and the fMRI validation procedure provided in this work should enable further development of deep linear models optimized for different types of real-world applications in biomedical imaging, with DELMAR/Denoise/Mapping as the current best algorithm for fMRI hierarchical functional connectivity mapping. Furthermore, using in-vivo rsfMRI MBME data, we validated our previous theoretical analyses (Zhang et al., 2020b). Namely, due to its independence constraints, ICA cannot reveal more components than DELMAR.

Besides, in this initial exploratory work on spatially hierarchical BCNs using MBME fMRI, several derived deep BCNs, i.e., identified third or fourth-layer BCNs, are consistent with known interactions between low-level BCNs from the established literature for resting-state fMRI. For example, the SN is known to modulate the anticorrelated connectivity of the DMN and the ECN (Menon et al., 2023), hence the linkage of their nodes into a single higher-level network (Figure 10, 6th row). The functional coupling of vision networks with the DAN shown in Figure 10 (4th row) is also well known, given the role that the DAN plays in visual attention and eye movements (Vossel et al., 2014). Future neuroscientific studies will be required to empirically validate the deep features of these methods using demographic, clinical, cognitive, behavioral, and/or electrophysiological data. Notably, since these deep linear models do not require extensive training datasets nor specialized computing infrastructure, they can be easily applied to clinical research with the potential to generate novel functional connectivity biomarkers of neurodevelopmental, neurodegenerative, and psychiatric disorders (Parkes et al., 2020), including for diagnosis, prognosis, and treatment monitoring. This is particularly significant given the recent observation that neuropathology and psychopathology often affect low-level network connectivity differently than high-level network connectivity. For example, many different psychiatric disorders have been found to decrease lower-order sensory and somatomotor network connectivity uniformly across patients (Elliott et al., 2018; Kebets et al., 2019) while increasing distinctiveness among patients in networks at higher levels of the hierarchy (Kaufmann et al., 2017; Parkes et al., 2020). In fMRI studies of mild traumatic brain injury (TBI), altered functional connectivity has been found early after concussion both within individual BCNs, such as the SN, DMN, and ECN, as well as between different BCNs (Palacios et al., 2017). Interactions of BCNs, such as those between the SN and the DMN, are thought to be especially important for outcomes after TBI and can be used to guide personalized treatment (Jilka et al., 2014; Li et al., 2019). Disordered coupling of the SN with the DMN and ECN has also been shown in mild cognitive impairment (Chand et al., 2017). Hence, prevalent neurological disorders such as head trauma and neurodegenerative diseases are thought to affect multiple levels of the human brain’s hierarchical organization. Such high-level interactions between the DMN, ECN, and SN can be investigated with deeper layers of these hierarchical linear models that integrate their spatially distinct gray matter nodes into a single larger-scale network, as shown in Figure 10 (refer to the top row). These examples show how more principled data-driven characterization of this hierarchy, particularly at its higher levels, holds great promise for providing clinically actionable biomarkers of neurological and psychiatric diseases.

Lastly, three potential shortcomings of the current work could be that the ground truth templates for testing the first and second-layer networks were generated using conventional shallow ICA (Smith et al., 2009), which is currently the most widely accepted technique for data-driven analysis of functional connectivity. Also, DELMAR/Denoise/Mapping cannot extract sub-networks, i.e., a minor-scale network including isolated regions from canonical BCNs, rather than meta-networks since it is designed as a dimensional reduction method. Therefore, it cannot perform subdivision of previous features in the deeper layers. Based on recent results reported by Wylie et al. (2021), higher-order ICA can subdivide shallow features into several sub-components.

## 5 Conclusion

To summarize, the benefits of DELMAR/Denoise/Mapping gain importance as the spatial and temporal resolution and sensitivity of fMRI continue to increase with improved MR imaging hardware and pulse sequences. Furthermore, DELMAR offers several advantages in detecting the hierarchical and overlapping organization of BCNs compared to previously described methods for mapping functional connectivity in a data-driven manner, such as ICA, SDL, DCAE, and DBN (Calhoun et al., 2001; Lv et al., 2015a,b; Seo et al., 2018; Zhang et al., 2020a,b; Hinton and Salakhutdinov, 2006; Hinton et al., 2012). DELMAR does not have the constraints of spatial independence that ICA has (Calhoun et al., 2001; Mckeown and Sejnowski, 1998; Zhang et al., 2019). Since DELMAR can reveal extensively overlapped functional brain networks (Zhang et al., 2020b) and estimates the vital hyperparameters automatically, it is easy to leverage the number and size of each layer, i.e., dictionary size. Other peer methods, such as ICA, SDL, NMF, and DNN, require manual hyperparameter tuning. Moreover, compared to DNNs, such as DBN, DCAE, and RBM, DELMAR has several advantages: (a) fewer training samples, e.g., the ability to reconstruct a single individual’s scan; (b) fewer extensive computational resources; (c) guaranteed convergence to a unique fixed point; and (d) automatic hyperparameter estimation. In this research, our results demonstrate the benefits of DELMAR/Denoise/Mapping for MBME imaging (Boyacioğlu et al., 2015; Cohen et al., 2020). Moreover, it can further benefit from the advent of even faster and higher-resolution SLice Dithered Enhanced Resolution Simultaneous MultiSlice (SLIDER-SMS) fMRI in the future (Vu et al., 2018). Continuously improved fMRI sensitivity and spatial resolution will enable mesoscale functional imaging that supports more shallow components of the deep linear learning method to reveal high-level networks and/or subnetworks of the spatially independent BCN used in Wylie et al. (2021). This will also permit the use of deep learning methods to extract more levels of the hierarchy of functional connectivity. Whereas many widely used methods for performing time-varying fMRI analysis are heuristic rather than data-driven, such as those with arbitrary time windows (Iraji et al., 2020), advances in fMRI temporal resolution can be combined with deep linear models that perform joint spatiotemporal decomposition for principled unsupervised dynamic functional connectivity mapping that reveals ever more of the human brain’s hierarchical organization. Notably, this work currently centers on the linear assumption that each meta-BCN is a linear combination of multiple canonical BCNs, i.e., BCNs identified from shallow layers of a deep learning method. Although this approach effectively captures essential hierarchy, advancing neuroscience increasingly reveals that more complex integrations, e.g., nonlinear combination, are needed to comprehensively capture hierarchical brain functionality. For example, isolated precuneus from DMN usually reflects the impairment of Alzheimer’s Disease on brain functionality (Billette et al., 2022). Therefore, we aim to further push beyond linear assumption, advancing our understanding of brain functionality through innovative computational frameworks that further deepen and expand our insights in understanding of brain functionality.

## Data Availability

Publicly available datasets were analyzed in this study. This data can be found here: https://openfmri.org/dataset/ds000216/.
