# Supplementary material for: Deep linear matrix approximate reconstruction with integrated BOLD signal denoising reveals reproducible hierarchical brain connectivity networks from multiband multi-echo fMRI
Source: Front Neurosci. 2025 Apr 16;19:1577029. doi: 10.3389/fnins.2025.1577029 (PMC12040835; doi:10.3389/fnins.2025.1577029)
Supplement: Supplementary file 1 [file Data_Sheet_1.pdf]

Supplementary Material

1 Supplementary Figures and Tables

1.1 1.1 Supplementary Tables

Table S1. All abbreviations of BCNs in Methodological Validation

Name/Number	Abbreviation	Name/Number	Abbreviation
Primary Visual Network/1	VIS-1	Auditory Network/7	AUD
Perception Visual Shape Network/2	VIS-2	Executive Control Network/8	ECN
Perception Visual Motion Network/3	VIS-3	Left Frontoparietal Network/9	FP-L
Default Mode Network/4	DMN	Right Frontoparietal Network/10	FP-R
Brainstem & Cerebellum Network/5	B/C	Dorsal Attention Network/11	DAN
Sensorimotor Network/6	SM	Salience Network/12	SN

Table S2. Important Hyperparameter/Important Parameters Settings of ME-ICA & DELMAR as well as DELMAR/Denoise/Mapping on MBME fMRI Data

Hyperparameters/Parameters	ME-ICA & DELMAR	DELMAR/Denoise/Mapping
Denoise/1st layer	370/382	300
1st / 2nd layer	96	72
2nd / 3rd layer	24	18
3rd / 4th layer	6	6
Number of Iterations	10,000*	10,000*
Learning Rate	0.01*	0.01*

1.2 Supplementary Figures

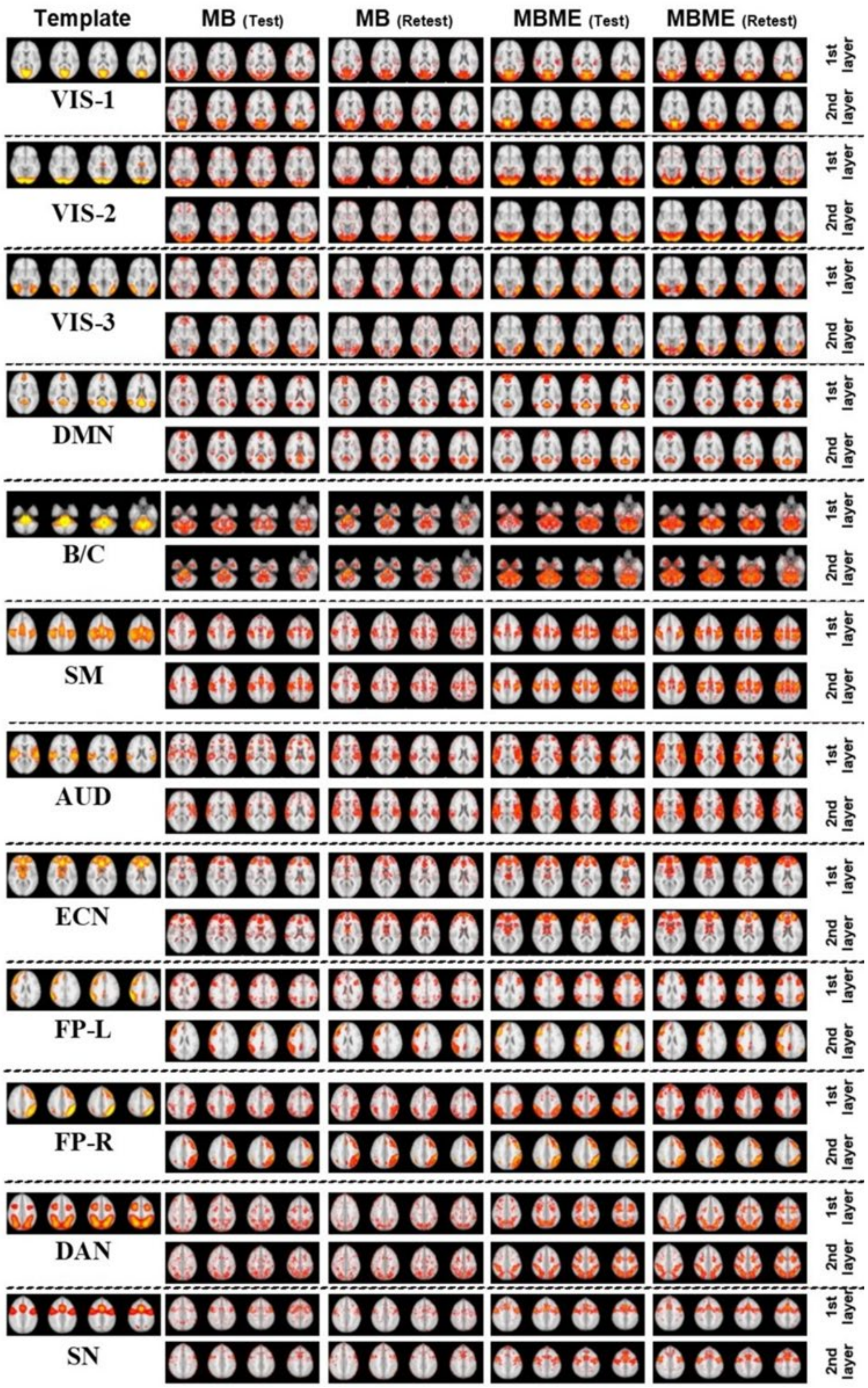

Supplementary Figure S1. Qualitative comparison of identified twelve 1st and 2nd layer conventional networks via MB and MBME fMRI, with canonical templates in the first column (please refer to Table S1 for detailed information of network #1-#12 and their abbreviations).

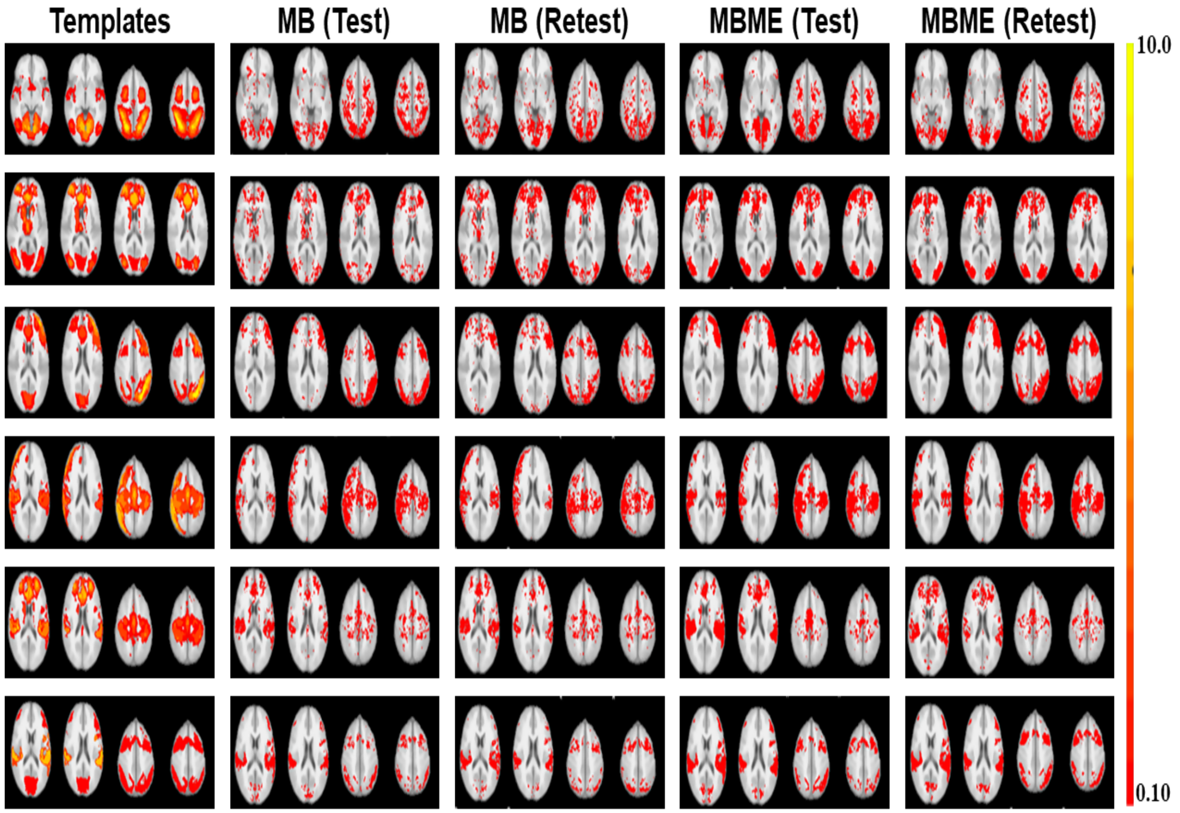

Supplementary Figure S2. Qualitative comparison of other six meta-BCNs identified via *DELMAR* at the third layer from test vs. retest MB and MBME fMRI data.

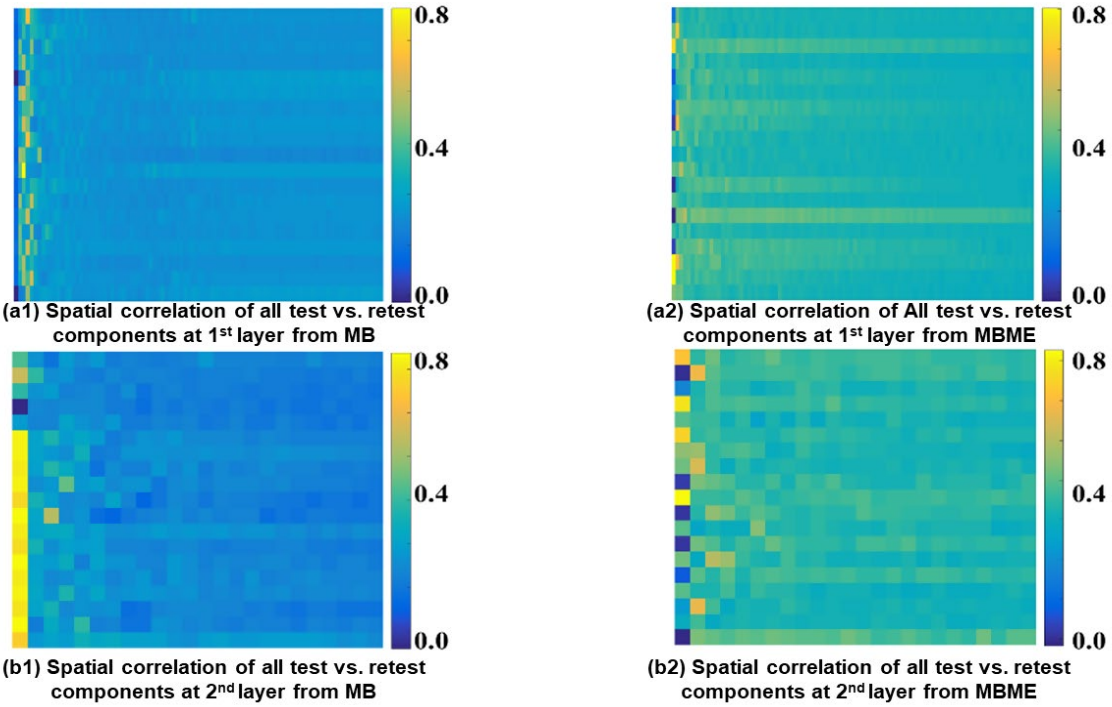

Supplementary Figure S3. Quantitative comparison of all BCNs identified via *DELMAR* at the first and second layers of *DELMAR* from test vs. retest MB and MBME fMRI data.
